# Runge–Kutta time semidiscretizations of semilinear PDEs with non-smooth data

**DOI:** 10.1007/s00211-015-0776-8

**Published:** 2015-11-17

**Authors:** Claudia Wulff, Chris Evans

**Affiliations:** Department of Mathematics, University of Surrey, Guildford, GU2 7XH UK

**Keywords:** 65J08, 65J15, 65M12, 65M15

## Abstract

We study semilinear evolution equations $\frac{\mathrm dU}{\mathrm dt}=AU+B(U)$ posed on a Hilbert space $\mathcal Y$, where *A* is normal and generates a strongly continuous semigroup, *B* is a smooth nonlinearity from $\mathcal Y_\ell = D(A^\ell )$ to itself, and $\ell \in I \subseteq [0,L]$, $L \ge 0$, $0,L \in I$. In particular the one-dimensional semilinear wave equation and nonlinear Schrödinger equation with periodic, Neumann and Dirichlet boundary conditions fit into this framework. We discretize the evolution equation with an A-stable Runge–Kutta method in time, retaining continuous space, and prove convergence of order $O(h^{p\ell /(p+1)})$ for non-smooth initial data $U^0\in \mathcal Y_\ell$, where $\ell \le p+1$, for a method of classical order *p*, extending a result by Brenner and Thomée for linear systems. Our approach is to project the semiflow and numerical method to spectral Galerkin approximations, and to balance the projection error with the error of the time discretization of the projected system. Numerical experiments suggest that our estimates are sharp.

## Introduction

We study the convergence of a class of A-stable Runge–Kutta time semidiscretizations of the semilinear evolution equation

1.1 $$\begin{aligned} \frac{\mathrm dU}{\mathrm dt} = AU + B(U) \end{aligned}$$

for non-smooth initial data $U(0)=U^0$. In the examples we have in mind (1.1) is a partial differential equation (PDE). We assume that (1.1) is posed on a Hilbert space $\mathcal Y$, *A* is a normal linear operator that generates a strongly continuous semigroup, and that *B* is smooth on a scale of Hilbert spaces $\{\mathcal Y_\ell \}_{\ell \in I}$, $I \subseteq [0,L]$, $0,L \in I$, as detailed in condition (B) below. Here $\mathcal Y_\ell =D(A^\ell )\subseteq \mathcal Y$, $\ell \ge 0$. Note that condition (B) depends on both, the smoothness properties of the nonlinearity *B*(*U*) and the boundary conditions. Under these assumptions the class of equations we consider includes the semilinear wave equation and the nonlinear Schrödinger equation in one spatial dimension with periodic, Neumann and Dirichlet boundary conditions (see Examples 2.3–2.8 below). For an example in three space dimensions see Example 5.4. We discretize (1.1) in time by an *A*-stable Runge Kutta method; the condition of *A*-stability ensures that the numerical method is well-defined on $\mathcal Y$, and is satisfied by a large class of methods including the Gauss–Legendre collocation methods.

Discretizing in time while retaining a continuous spatial parameter means that we consider the numerical method as a nonlinear operator on the infinite dimensional space $\mathcal Y$. This leads to several technicalities, in particular existence results for the numerical method $\Psi ^h$ as well as the semiflow $\Phi ^t$ and regularity of solutions in both cases are required to ensure convergence results analogous to the finite dimensional case. In [15], existence and regularity of the semiflow of (1.1) on a scale of Hilbert spaces, corresponding results for the numerical method, and full order convergence of the time semidiscretization for sufficiently smooth data are studied in detail. We review the relevant results in Sects. 2 and 3.

**Fig. 1:**
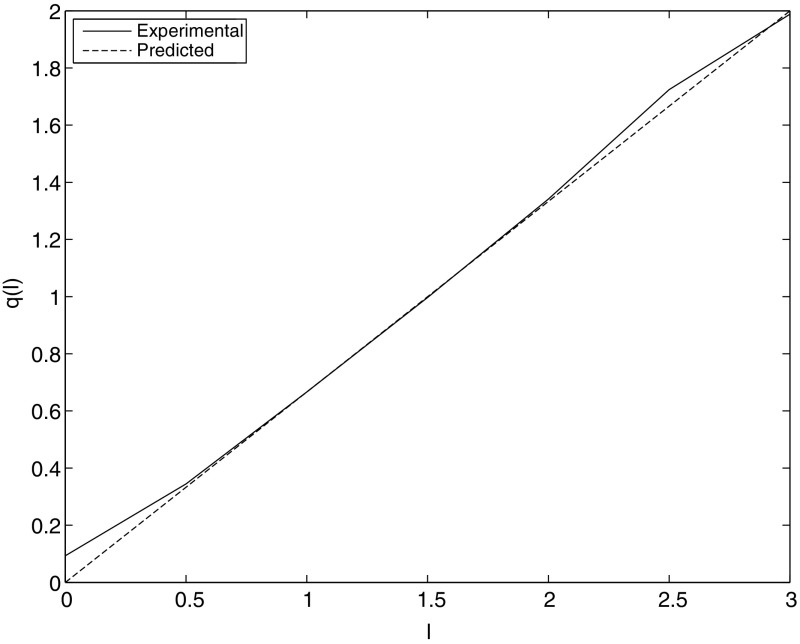
Plot of a numerical estimate of $q(\ell )$ against $\ell$ for the implicit midpoint rule applied to the semilinear wave equation, with the prediction of Theorem 5.3 for comparison

In this paper we consider the effect of non-smooth data on the order of convergence of the time semidiscretization in this setting. We consider an *A*-stable Runge–Kutta method of classical order *p* applied to the problem (1.1) with initial data $U^0\in \mathcal Y_\ell$, $\ell \in I$. The main result we give here, Theorem 5.3, shows that we can expect order of convergence $\mathcal O(h^q)$ where $q(\ell ) = p\ell /(p+1)$ for $0\le \ell < p+1$. This corresponds closely with numerical observation, cf. Fig. 1. Given a time $T>0$ we prove the above order of convergence for the time-semidiscretization up to time *T* for any solution *U*(*t*) of (1.1) with a given $\mathcal Y_\ell$ bound. Here $\ell >0$ is such that $\ell -k \in I$ for $k=1,\ldots , \lfloor \ell \rfloor$ (the greatest integer $\le \ell$). It is shown in [15] that for $\ell \ge p+1$ we have full order of convergence $\mathcal O(h^p)$.

The reduction in order of the method from *p* to *q* for $\ell <p+1$ is caused by the occurrence of unbounded operators in the Taylor expansion of the one-step error coefficient. Our approach is to apply a spectral Galerkin approximation to the semiflow of the evolution equation (1.1), and to discretize the projected evolution equation in time. This allows us to bound the size of the local error coefficients in terms of the accuracy of the projection. By balancing the projection error with the growth of the local error coefficients we obtain the estimates of our main result, Theorem 5.3.

Related results include those of Brenner and Thomée [3], who consider linear evolution equations $\dot{U}=AU$ in a more general setting, namely posed on a Banach space $\mathcal X$, where *A* generates a strongly continuous semigroup $e^{tA}$ on $\mathcal X$. They show $O(h^q)$ convergence of A-acceptable rational approximations of the semigroup for non-smooth initial data $U^0\in D(A^\ell )$, $\ell = 0,\ldots ,p+1$, with $q=q(\ell )= p \ell /(p+1)$ as above, if $\ell > (p+1)/2$ (when $\ell \le (p+1)/2$ they prove convergence with order $q(\ell )< p \ell /(p+1)$). Kovács [9] generalizes this result to certain intermediate spaces with arbitrary $\ell \in [0,p+1]$ and also provides sufficient conditions for when $q=q(\ell )= p \ell /(p+1)$ for all $\ell \in [0,p+1]$ (which are satisfied in our setting).

For splitting methods, where the linear part of the evolution equation is evaluated exactly, a higher order of convergence has been obtained for specific choices of $\ell$ and specific evolution equations in [6] and [13], see also Example 5.4 below. While splitting methods are very effective for simulating evolution equations for which the linear evolution $\mathrm e^{tA}$ can easily be computed explicitly, Runge–Kutta methods are still a good choice when an eigen-decomposition of *A* is not available, for example for the semilinear wave equation in an inhomogeneous medium, see Example 2.7. Moreover, the simplest example of a Gauss–Legendre Runge–Kutta method, the implicit mid point rule, appears to have some advantage over split step time-semidiscretizations for the computation of wave trains for nonlinear Schrödinger equations because the latter introduce an artificial instability [18].

For Runge–Kutta time semidiscretizations of dissipative evolution equations, where *A* is sectorial, a better order of convergence can be obtained, see [10] for the linear case and [11, 12] and references therein for the semilinear case.

Note that our approach is different from the approach of [11, 12]. In [11, 12] some smoothness of the continuous solution is assumed and from that a (fractional) order of convergence is obtained, using the variation of constants formula. The order of convergence obtained in [11, 12] is in general lower than in the linear case (where full order of convergence is obtained in the parabolic case [10]), but no extra assumptions on the nonlinearity *B*(*U*) of the PDE are made. In particular in [12, Theorems 4.1 and 4.2] the existence of $(p_s+2)$ time derivatives of the continuous solution *U*(*t*) of a semilinear parabolic PDE (1.1) is assumed, where $p_s$ is the stage order of the method. This assumption is then used to estimate the error of the numerical approximation of the inhomogenous part of the variation of constants formula. Here the stage order $p_s$ comes into play. Note that if the nonlinearity *B*(*U*) of the evolution equation (1.1) only satisfies the standard assumption rather than our assumption (B), i.e., is smooth on $\mathcal Y$ only (so that the Hilbert space scale is trivial with $L=0$) then the existence of $U'(t)$ can be guaranteed for $U^0 \in \mathcal Y_1$ by semigroup theory [17], but it is not clear whether higher order time derivatives of the solution *U*(*t*) of (1.1) exist as assumed in [12]—therefore in [12] also time-dependent perturbations of (1.1) are considered. In this paper we instead take the approach of making assumptions (namely condition (B) on the nonlinearity *B*(*U*) of the evolution equation and the condition that $U^0 \in \mathcal Y_\ell$) which are straightforward to check and guarantee the existence of the time derivatives of the continuous solution *U*(*t*) up to order $k\le \ell$. We then obtain an order of convergence $O(h^{p\ell /(p+1)})$ of the Runge–Kutta discretization which is identical to the order of convergence in the linear case [3, 9]. In [11, Theorem 2.1] some smoothness of the inhomogeneity of the PDE is obtained from the smoothing properties of parabolic PDEs, and this is used to prove an order of convergence $h\log h$, without the assumption of the existence of higher time derivatives of the continuous solution *U*(*t*). Here we do not consider parabolic PDEs, so that we cannot use this strategy.

Alonso-Mallo and Palencia [2] study Runge–Kutta time discretizations of inhomogeneous linear evolution equations where the linear part creates a strongly continuous semigroup. Similarly as in [12] they obtain an order of convergence depending on the stage order $p_s$ of the Runge–Kutta method. They assume the continuous solution *U*(*t*) to be $(p+1)$-times differentiable in *t*, but in their context the condition $U(t) \in D(A^{p-p_s})$, where *p* is the order of the numerical method, is in general not satisfied due to the inhomogeneous terms in the evolution equation, and this leads to a loss in the order of convergence compared to our results. Note that in our setting, due to our condition (B) on the nonlinearity, provided $U(0) \in \mathcal Y_{p+1}$ we have $U(t) \in D(A^{p+1}) = \mathcal Y_{p+1}$ and *U*(*t*) is $p+1$ times differentiable in *t* (in the $\mathcal Y$ norm) and so we get full order of convergence in this case (see [15]). Calvo et al. [4] study Runge–Kutta quadrature methods for linear evolution equations $\dot{U}(t) = A(t)U(t)$ which are well-posed and prove full order convergence if the continuous solution *U*(*t*) has $p+1$ time derivatives; they also obtain fractional orders of convergence as in [3] for solutions $U(t) \in \mathcal Y_\ell$ with $\ell <p+1$.

We proceed as follows: in Sect. 2 we introduce the class of semilinear evolution equations that we consider in this paper, give some examples, review existence and regularity results of [15, 17] for the semiflow, and adapt them to the case of non-integer $\ell$. In Sect. 3 we introduce a class of *A*-stable Runge–Kutta methods. We review existence and regularity of these methods when applied to the semilinear evolution equation (1.1) and a convergence result for sufficiently smooth initial data from [15]. In Sect. 4 we study the stability of the semiflow and numerical method under spectral Galerkin truncation, and establish estimates for the projection error. Lemma 4.2 and 4.3 are established in [16] for integer values of $\ell$; for completeness we review the proofs, which also work for non-integer $\ell$. In Sect. 5 we prove our main result on convergence of *A*-stable Runge–Kutta discretizations of semilinear evolution equations for non-smooth initial data.

## Semilinear PDEs on a scale of Hilbert spaces

In this section we introduce a suitable functional setting for the class of equations we subsequently study. We review results from [15, 17] on the local well-posedness and regularity of solutions of (1.1) and give examples.

For a Hilbert space $\mathcal X$ we let

$$\begin{aligned} \mathcal B^R_{\mathcal X}(U^0)=\{U\in \mathcal X:||U-U^0 ||_{\mathcal X}^{}\le R\} \end{aligned}$$

be the closed ball of radius *R* around $U^0$ in $\mathcal X$. We make the following assumptions on the semilinear evolution equation (1.1):

(A) *A* is a normal linear operator on $\mathcal Y$ that generates a strongly continuous semigroup of linear operators $e^{tA}$ on $\mathcal Y$ in the sense of [17].

It follows from assumption (A) that there exists $\omega \in \mathbb R$ with

2.1 $$\begin{aligned} \mathfrak {Re}({\text {spec}}\ (A))\le \omega , \quad ||e^{tA} ||_{\mathcal Y\rightarrow \mathcal Y}^{}\le e^{\omega t}, \end{aligned}$$

see [17]. In light of (A) we define the continuous scale of Hilbert spaces $\mathcal Y_\ell = D(A^\ell )$, $\ell \ge 0$, $\mathcal Y_0=\mathcal Y$. Thus the parameter $\ell$ is our measure of smoothness of the data. For $m>0$ we define $\mathbb P_m$ to be the spectral projection of *A* to ${\text {spec}}\ (A) \cap \mathcal B^m_\mathbb C(0)$, let $\mathbb Q_m={\text {id}}-\mathbb P_m$ and set $\mathbb P=\mathbb P_1$, $\mathbb Q= {\text {id}}- \mathbb P$. We endow $\mathcal Y_\ell$ with the inner product

2.2 $$\begin{aligned} \langle U_1,U_2 \rangle _{\mathcal Y_\ell }^{}=\langle \mathbb PU_1,\mathbb PU_2 \rangle _{\mathcal Y}^{} + \langle |A|^\ell \mathbb QU_1,|A|^\ell \mathbb QU_2 \rangle _{\mathcal Y}^{}, \end{aligned}$$

which implies

2.3 $$\begin{aligned} \Vert A^\ell \Vert _{\mathcal Y_\ell \rightarrow \mathcal Y} \le 1. \end{aligned}$$

We deduce from assumption (A) that for $u\in \mathcal Y$, $\lim _{m\rightarrow \infty }\mathbb P_mu=u$, and from (2.2) the estimates

2.4 $$\begin{aligned} ||A^\ell \mathbb P_m U ||_{\mathcal Y}^{}\le m^\ell ||\mathbb P_m U ||_{\mathcal Y}^{},\quad \Vert \mathbb P_m \Vert _{ \mathcal Y_\ell \rightarrow \mathcal Y_{\ell +k}} \le m^k, \quad ||\mathbb Q_m U ||_{\mathcal Y}^{}\le m^{-\ell }||U ||_{\mathcal Y_\ell }^{} \end{aligned}$$

for $$\ell \ge 0$$, $$k\ge 0$$, $$m\ge 1$$.

### *Remark 2.1*

When $\ell$ lies in a discrete set such as $\mathbb N_0$, for $\ell >0$ often the inner product

2.5 $$\begin{aligned} \langle U_1,U_2 \rangle _{\ell }^{} =\langle U_1,U_2 \rangle _{\mathcal Y}^{}+\langle A^\ell U_1, A^\ell U_2 \rangle _{\mathcal Y}^{} \end{aligned}$$

is used on $\mathcal Y_\ell$ instead of (2.2). For $\ell =0$, for consistency, one defines $\langle U_1,U_2 \rangle _{0}^{} =\langle U_1,U_2 \rangle _{\mathcal Y}^{}$. The reason why we do not use this inner product here is that (2.2) is continuous in $\ell$ as $\ell \rightarrow 0$, but the graph inner product (2.5) is not: we have $\lim _{\ell \rightarrow 0} \langle U_1,U_2 \rangle _{\ell }^{} = 2\langle U_1,U_2 \rangle _{\mathcal Y}^{}= 2\langle U_1,U_2 \rangle _{0}^{}$.

To formulate our second assumption, on the nonlinearity *B*, we introduce the following notation: for Banach spaces $\mathcal X$, $\mathcal Z$, we denote by $\mathcal E^i(\mathcal X,\mathcal Z)$ the space of *i*-multilinear bounded mappings from $\mathcal X$ to $\mathcal Z$. For $\mathcal U\subseteq \mathcal X$ we write $\mathcal C_{{\text {b}}}^k(\mathcal U,\mathcal Z)$ to denote the set of *k* times continuously differentiable functions $F :{\text {int}}\mathcal U\rightarrow \mathcal Z$ such that *F* and its derivatives $\mathrm D^i F$ are bounded as maps from the interior ${\text {int}}\mathcal U$ of $\mathcal U$ to $\mathcal E^i(\mathcal X,\mathcal Z)$ and extend continuously to the boundary of ${\text {int}}\mathcal U$ for $i \le k$. We set $\mathcal C_{{\text {b}}}(\mathcal U,\mathcal Z)= \mathcal C_{{\text {b}}}^0(\mathcal U,\mathcal Z)$. Note that if $\dim \mathcal X=\infty$, there are examples of continuous functions $F:\mathcal U\rightarrow \mathcal Z$ where $\mathcal U$ is closed and bounded, which do not lie in $\mathcal C_{{\text {b}}}(\mathcal U,\mathcal Z)$, see e.g. [15, Remark 2.3]. In the following for $\ell \in \mathbb R$ let $\lfloor \ell \rfloor$ be the largest integer less than or equal to $\ell$ and $\lceil \ell \rceil$ be the smallest integer greater or equal to $\ell$. Moreover for $R>0$ and $\ell \ge 0$ we abbreviate

2.6 $$\begin{aligned} \mathcal B_\ell ^{R} = \mathcal B_{\mathcal Y_\ell }^R(0). \end{aligned}$$

We are now ready to formulate our condition on the nonlinearity *B*(*U*) of (1.1).

(B) There exists $L\ge 0$, $I \subseteq [0,L]$, $0,L \in I$, $N\in \mathbb N$, $N > \lceil L\rceil$, such that $B\in \mathcal C_{{\text {b}}}^{N- \lceil \ell \rceil }(\mathcal B^R_\ell ;\mathcal Y_\ell )$ for all $\ell \in I$ and $R>0$.

We denote the supremum of $$B:\mathcal B_\ell ^R\rightarrow \mathcal Y_\ell $$ as $$M_\ell [R]$$ and the supremum of its derivative as $$M'_\ell [R]$$, and set $$M[R] =M_0[R] $$ and $$M'[R] =M'_0[R]$$. Moreover we define

2.7 $$\begin{aligned} I^-:= \{ \ell \in I, \ell -k\in I, k =1,\ldots , \lfloor \ell \rfloor \}. \end{aligned}$$

We seek a solution $U(\cdot )\in \mathcal C([0,T];\mathcal Y_\ell )$ of (1.1) for some $T>0$, $\ell \in I$, with initial data $U(0)=U^0\in \mathcal Y_\ell$, and write $\Phi ^t(U^0)\equiv \Phi (U^0,t)\equiv U(t)$. The following result is an extension of Theorem 2.4 of [15], see also [17], to non-integer $\ell$ and provides well-posedness and regularity of the semiflow $\Phi ^t$ under suitable assumptions.

### **Theorem 2.2**

(Regularity of the semiflow) Assume that the semilinear evolution equation (1.1) satisfies (A) and (B). Let $R>0$. Then there is $T_*>0$ such that there exists a semiflow $\Phi$ which satisfies

2.8a $$\begin{aligned} \Phi ^t\in \mathcal C_{{\text {b}}}^{N}(\mathcal B_0^{R/2};\mathcal B^R_0) \end{aligned}$$

with uniform bounds in $t\in [0,T_*]$. Moreover if $\ell \in I^-$ and $k\in \mathbb N_0$ satisfies $k \le \ell$, then

2.8b $$\begin{aligned} \Phi (U) \in \mathcal C_{{\text {b}}}^k([0,T_*];\mathcal B^R_0) \end{aligned}$$

with uniform bounds in $U \in \mathcal B_\ell ^{R/2}$. The bounds on $T_*$ and $\Phi$ depend only on *R*, $\omega$ from (2.1), and the bounds afforded by assumption (B) on balls of radius *R*.

### *Proof*

The proof of (2.8) is an application of a contraction mapping theorem with parameters to the map

2.9 $$\begin{aligned} \Pi (W,U,T) =e^{tTA}U + \int _0^t e^{T(t-\tau )A}B(W(\tau )) \mathrm d\tau , \end{aligned}$$

on the scale of Banach spaces $\mathcal Z_{\ell } = \mathcal C_{{\text {b}}}([0,1];\mathcal Y_{\ell })$, $\ell \in I$, where we define $\mathcal Z:= \mathcal Z_0$. The solution $W(U,T)(t) =\Phi ^{tT}(U)$ of (1.1) is obtained as a fixed point of (2.9) for $U \in \mathcal B^{R/2}_\mathcal Y(0)$ as in [15]. Here $\Pi :\mathcal B^{R}_{\mathcal Z}(0) \times \mathcal B^{R/2}_\mathcal Y(0)\times [0,T_*]\rightarrow \mathcal Z$. In order to apply the contraction mapping theorem we first check that $\Pi (W,\cdot ,\cdot )$ maps $\mathcal B^{R}_{\mathcal Z}( 0)$ to itself: For $U\in \mathcal B^{R/2}_\mathcal Y(0)$ we have

2.10 $$\begin{aligned} \Vert \Pi (W,U,T) \Vert _{\mathcal Z}&\le \max _{\tau \in [0,1]} \Vert \mathrm e^{\tau TA} U\Vert _{\mathcal Y} + T \mathrm e^{\omega T} M_0[R] \nonumber \\&\le \mathrm e^{\omega T}R/2 + T \mathrm e^{\omega T} M_0[R] \le R \end{aligned}$$

for $T\in [0,T_*]$ and $T_*$ small enough. So $\Pi$ maps $\mathcal B^{R}_{\mathcal Z}( 0)$ to itself. Moreover for sufficiently small $T_*$ there is $c\in [0,1)$ such that $\Vert \mathrm D\Pi (W,U,T)\Vert _{\mathcal Z\rightarrow \mathcal Z}\le c$ for all $W \in \mathcal B^R_{\mathcal Z}(0)$, $U \in \mathcal B^{R/2}_\mathcal Y(0)$ and $T \in [0,T_*]$ so that $\Pi$ is a contraction. Hence, $W\in \mathcal C_{{\text {b}}}(\mathcal B^{R/2}_{\mathcal Y}( 0) \times [0,T_*];\mathcal B^{R}_{\mathcal Z}( 0))$ with *N* derivatives in the first component. This proves statements (2.8a) and also $\Phi (U) \in \mathcal C_{{\text {b}}}^k([0,T_*];\mathcal B_0^R)$ in the case $k=0$.

For $k \in \mathbb N$, $k\le \ell$ it follows from the fact $\ell \in I^-$ that the above argument applies with $\mathcal Y$ replaced by $\mathcal Y_{\ell -j}$, $j=0,\ldots , k$. Hence there is some $T_*>0$ such that $\Phi \in \mathcal C_{{\text {b}}}(\mathcal B^{R/2}_{\ell -j}\times [0,T_*];\mathcal B^{R}_{\ell -j})$ for $j=0,\ldots , k$. As detailed in [15] for $U \in \mathcal B^{R/2}_{\ell }$ the *t* derivatives up to order *k* can then be obtained by implicit differentiation of $\Pi (W(U,T),U,T)=W(U,T)$ with $\Pi$ defined above which implies that $\Phi (U) \in \mathcal C_{{\text {b}}}^k([0,T_*];\mathcal B^R_0)$ for $k\le \ell$ with uniform bounds in $U \in \mathcal B^{R/2}_{\ell }$. $\square$

Note that this theorem extends to mixed (*U*, *t*) derivatives which are, however, in general only strongly continuous in *t*, see [15] for details. For our purposes in this paper the above theorem is sufficient.

### *Example 2.3*

(*Semilinear wave equation, periodic boundary conditions*) Consider the semilinear wave equation

2.11 $$\begin{aligned} \partial _{tt}u = \partial _{xx} u - V'(u) \end{aligned}$$

on $[0,2\pi ]$ with periodic boundary conditions. Writing $v=\partial _tu$ and $U=(u,v)^T$. Equation (2.11) takes the form (1.1) where

2.12 $$\begin{aligned} A =\mathbb Q_0 \tilde{A}, \quad \tilde{A} = \begin{pmatrix} 0 &{}\quad {\text {id}}\\ \partial ^2_x &{}\quad 0 \end{pmatrix}, \qquad B(U) = {0 \atopwithdelims ()-V'(u)} + \mathbb P_0 \tilde{A} U. \end{aligned}$$

Here $\mathbb P_0$ is the spectral projector of $\tilde{A}$ to the eigenvalue 0. Since the Laplacian is diagonal in the Fourier representation with eigenvalues $-k^2$ for $k\in \mathbb Z$, the eigenvalue problem for *A* separates into $2\times 2$ eigenvalue problems on each Fourier mode, and it is easy to see that the spectrum of *A* is given by

$$\begin{aligned} {\text {spec}}\ A = \{ \mathrm ik :k \in \mathbb Z\} {\setminus } \{0\}. \end{aligned}$$

Note that $\mathbb P_0 \tilde{A}$ has a Jordan block and is hence included with the nonlinearity *B*. We denote the Fourier coefficients of a function $u \in \mathcal L^2([0,2\pi ];\mathbb R^d)$ by $\hat{u}_k$, so that

2.13 $$\begin{aligned} u(x) = \frac{1}{\sqrt{2\pi }} \sum _{k\in \mathbb Z} \hat{u}_k \, {\mathrm e}^{{\mathrm i}kx}. \end{aligned}$$

Then the Sobolev space $\mathcal H_{\ell }([0,2\pi ];\mathbb R^d)$ is the Hilbert space of all $u \in \mathcal L^2([0,2\pi ];\mathbb R^d)$ for which

$$\begin{aligned} ||u ||_{\mathcal H_{\ell }}^{2} = \langle u , u \rangle _{\mathcal H_{\ell }} < \infty , \end{aligned}$$

where the inner product is given by

2.14 $$\begin{aligned} \langle u, v \rangle _{\mathcal H_{\ell }} = \langle \hat{u}_0, \hat{v}_0 \rangle _{\mathbb R^d}+ \sum _{k\in \mathbb Z} |k|^{2\ell } \, \, \langle \hat{u}_k , \hat{v}_k \rangle _{\mathbb R^d}. \end{aligned}$$

In the setting of the semilinear wave equation, we have

2.15 $$\begin{aligned} \mathcal Y_{\ell } = \mathcal H_{\ell +1}([0,2\pi ];\mathbb R) \times \mathcal H_{\ell }([0,2\pi ];\mathbb R), \end{aligned}$$

and the group $e^{tA}$ is unitary on any $\mathcal Y_{\ell }$. So (A) is satisfied. Moreover in this example, the inner product (2.2) on $\mathcal Y_\ell$ corresponds to the inner product defined via (2.14). If the potential $V:\mathbb R\rightarrow \mathbb R$ is analytic, then, by Lemma 2.9 a) below, the nonlinearity *B*(*U*) is analytic as map of $\mathcal Y_{\ell }$ to itself for any $\ell \ge 0$ and *B* and its derivatives are bounded on balls around 0. Hence assumption (B) holds for any $L\ge 0$ and $N> \lceil L \rceil$ with $I=[0,L]$.

### *Example 2.4*

(*Semilinear wave equation, non-analytic nonlinearity*) If $V\in \mathcal C^{N+2}(\mathbb R)$ then (B) holds with $I = [0,L]$ and $\lceil L\rceil < N$. To see this note that Lemma 2.9 c) applied to $f = V' \in \mathcal C^{N+1}(\mathbb R)$ ensures that $f \in \mathcal C_{{\text {b}}}^{N-\lfloor \ell \rfloor }(\mathcal B_{\mathcal H_{\ell +1}}^R; \mathcal H_{\ell })$ for all $R>0$ and therefore that (B) holds, noting that $\mathcal Y_\ell$ is as in (2.15). Here we abbreviated $\mathcal H_{\ell }:=\mathcal H_{\ell }([0,2\pi ];\mathbb R)$.

### *Example 2.5*

(*Semilinear wave equation, Dirichlet boundary conditions*) When endowed with homogeneous Dirichlet boundary conditions $u(t,0) = u(t,\pi )=0$ the linear part *A* of the semilinear wave equation (2.11) still generates a unitary group. In this case we have $\mathbb P_0=0$, $A=\tilde{A}$, and

$$\begin{aligned} \mathcal Y_\ell =D(A^\ell ) = \mathcal H^0_{\ell +1} ([0,\pi ];\mathbb R) \times \mathcal H_\ell ^0 ([0,\pi ];\mathbb R). \end{aligned}$$

Here $\mathcal H_\ell ^0 ([0,\pi ];\mathbb R) = D((-\Delta )^{\ell /2})$, where $\Delta$ denotes the Laplacian with Dirichlet boundary conditions. By [8] for $\ell \notin 2 \mathbb N_0+\frac{1}{2}$

$$\begin{aligned} \mathcal H_\ell ^0 ([0,\pi ];\mathbb R) \!=\! \left\{ u \!\in \! \mathcal H_\ell ([0,\pi ];\mathbb R): u^{(2j)}(0)\!=\!u^{(2j)}(\pi )\!=\!0\quad \text{ for }\quad 0\le 2j\!<\! \ell -\frac{1}{2} \right\} . \end{aligned}$$

If $V:\mathbb R\rightarrow \mathbb R$ is analytic and even so that $f=-V'$ satisfies the required boundary conditions, the conclusions of Lemma 2.9 a) apply to $f=-V'$ on the spaces $\mathcal H_{\ell +1}^0 ([0,\pi ];\mathbb R)$ and $\mathcal H_{\ell }^0 ([0,\pi ];\mathbb R)$, provided that $\ell +1\notin \frac{1}{2} + 2\mathbb N_0$ or $\ell \notin \frac{1}{2} + 2\mathbb N_0$, respectively. Since we need $-V'(u)$ to map from an open set of $\mathcal H_{\ell +1}^0 ([0,\pi ];\mathbb R)$ into $\mathcal H_{\ell }^0 ([0,\pi ];\mathbb R)$ it is sufficient to satisfy either of those two constraints on $\ell$, at least one of which is always true. So in this example condition (B) is satisfied with $I = [0,L]$ for any $L\ge 0$. Moreover the condition that *V* is even may be relaxed to the requirement that $V^{(2j+1)}(0)=0$ for $0\le 2j\le L+\frac{1}{2}$.

### *Example 2.6*

(*Semilinear wave equation, Neumann boundary conditions*) In the case of Neumann boundary conditions on $[0,\pi ]$, the operator $A=\tilde{A}$ from (2.12) is again skew-symmetric and has the same spectrum as in Example 2.3. In this case, $\mathcal Y_\ell = \mathcal H_{\ell +1}^{{\text {nb}}}( [0,\pi ];\mathbb R) \times \mathcal H_{\ell }^{{\text {nb}}}( [0,\pi ];\mathbb R)$. Here $\mathcal H_\ell ^{{\text {nb}}}([0,\pi ];\mathbb R) = D((-\Delta )^{\ell /2})$, where $\Delta$ now denotes the Laplacian with Neumann boundary conditions. Due to [8]

$$\begin{aligned} \mathcal H_{\ell }^{{\text {nb}}}([0,\pi ];\mathbb R) \!=\! \left\{ u \!\in \! \mathcal H_{\ell }([0,\pi ];\mathbb R) :u^{(2j+1)}(0) \!=\! u^{(2j+1)}(\pi )\!=\!0\quad \text {for}\quad 0\!\le \! 2 j <\ell -\frac{ 3}{2} \right\} , \end{aligned}$$

for $$\ell \notin 3/2+2\mathbb N_0$$. If $$V:\mathbb R\rightarrow \mathbb R$$ is analytic, then the conclusions of Lemma 2.9 a) apply to $$f=-V'$$ on the spaces $$\mathcal H_{\ell +1}^{{\text {nb}}}([0,\pi ];\mathbb R)$$ ($$\mathcal H_{\ell }^{{\text {nb}}}([0,\pi ];\mathbb R)$$) whenever $$\ell +1\notin \frac{3}{2} + 2\mathbb N_0$$ ($$\ell \notin \frac{3}{2} + 2\mathbb N_0$$). This follows from the fact that all terms in the sum obtained from computing $$\partial _x^{2j+1}f(u)$$ contain at least one odd derivative of *u* of order at most $$2j + 1$$, so that the required boundary conditions for *f* are satisfied. Hence Condition (B) is satisfied for any $$L\ge 0$$ with $$I = [0,L] $$.

### *Example 2.7*

(*A semilinear wave equation in an inhomogeneous material*) Instead of (2.11), let us consider the non-constant coefficient semilinear wave equation

$$\begin{aligned} \partial _{tt} u = \partial _x (a \, \partial _x u) + b \, u -V'(u) \end{aligned}$$

with periodic boundary conditions where $V\in \mathcal C^{N+2}(\mathbb R)$, $a,b \in \mathcal C_{{\text {b}}}^N([0,2\pi ];\mathbb R)$ are $2\pi$-periodic with $a(x) >0$ and $b(x) \le 0$ for $x \in [0,2\pi ]$. Then the conclusions of Example 2.4 apply.

### *Example 2.8*

(*Nonlinear Schrödinger equation*) Consider the nonlinear Schrödinger equation

2.16 $$\begin{aligned} \mathrm i\, \partial _t u = \partial _{xx} u + \partial _{\bar{u}} V(u,\bar{u}) \end{aligned}$$

on $[0,2\pi ]$ with periodic boundary conditions, where $V(u,\bar{u})$ is assumed to be analytic as a function in $u_1=\mathfrak {Re}\,(u)$ and $u_2=\mathfrak {I}\,(u)$. Setting $U =(u_1, u_2)$, we can write (2.16) in the form (1.1) with

2.17 $$\begin{aligned} A = \left( \begin{array}{c@{\quad }c} 0 &{} \partial ^2_x\\ -\partial _x^2 &{} 0 \end{array} \right) , \quad B(U) = \frac{1}{2} { \partial _{u_2} V \atopwithdelims ()- \partial _{u_1} V}. \end{aligned}$$

The Laplacian is diagonal in the Fourier representation (2.13) with eigenvalues $-k^2$ and $\mathcal L^2([0,2\pi ];\mathbb C)$-orthonormal basis of eigenvectors $\mathrm e^{\pm \mathrm ik x}/\sqrt{2\pi }$ where $k \in \mathbb Z$. Hence, the spectrum of *A* is given by

$$\begin{aligned} {\text {spec}}\ A = \{ -\mathrm ik^2 :k \in \mathbb Z\} \end{aligned}$$

and *A* is normal and generates a unitary group on $\mathcal L^2([0,2\pi ];\mathbb C)$ and, more generally, on every $\mathcal H_{\ell }([0,2\pi ];\mathbb C)$ with $\ell \ge 0$.

By Lemma 2.9 a) below the nonlinearity *B*(*U*) defined in (2.17) is analytic as map from $\mathcal H_{\ell }([0,2\pi ];\mathbb R^2)$ to itself for every $\ell >1/2$. Hence, assumption (B) holds for the nonlinear Schrödinger equation (2.16) for any $I=[0,L]$, $L\ge 0$ if we set $\mathcal Y_\ell = \mathcal H_{2\ell +\alpha }([0,2\pi ];\mathbb R^2)$ for $\alpha >1/2$.

When we equip the nonlinear Schrödinger equation (2.16) with Dirichlet (Neumann) boundary conditions we need to require that $\ell +\frac{\alpha }{2} \notin \mathbb N_0 + \frac{1}{4}$ ($\ell +\frac{\alpha }{2} \notin \mathbb N_0 + \frac{3}{4}$) and, for Dirichlet boundary conditions, we need the potential *V* to be even or satisfy $V^{(2j+1)}(0)=0$ for $0\le j< L+\alpha -\frac{1}{4}$. Here $I = [0,L] {\setminus } (\mathbb N_0 + \frac{1}{4}-\frac{\alpha }{2})$ for Dirichlet boundary conditions and $I = [0,L] {\setminus } (\mathbb N_0 + \frac{3}{4}-\frac{\alpha }{2})$ for Neumann boundary conditions.

The nonlinearities of the PDEs in the above examples are superposition operators $f :\mathcal H_{\ell }([0,2\pi ];\mathbb R^d) \rightarrow \mathcal H_{\ell }([0,2\pi ];\mathbb R^d)$ of smooth functions $f:D \subseteq \mathbb R^d \rightarrow \mathbb R^d$ or restrictions of such operators to spaces encorporating boundary conditions. To prove that these superposition operators satisfy assumption (B) we have employed the following lemma. Part a) of this lemma has already been stated in slightly different form in [7, 14], and parts b) and c) follow from [15].

### **Lemma 2.9**

(Superposition operators) Let $\Omega \subseteq \mathbb R^n$ be an open set satisfying the cone property.

- Let $\rho >0$ and let $f :\mathcal B_{\mathbb C^d}^\rho \rightarrow \mathbb C^d$ be analytic. If $\Omega$ is unbounded assume $f(0)=0$. Then *f* is also analytic as a function from $\mathcal B_{\mathcal H_\ell }^R$ to $\mathcal H_{\ell }:= \mathcal H_{\ell }(\Omega ;\mathbb C^d)$ for every $\ell > n/2$ and $R\le \rho /c$ with *c* from (2.19) below. Moreover $f:\mathcal B_{\mathcal H_\ell }^R \rightarrow \mathcal H_\ell$ and its derivatives up to order *N* are bounded with *N*-dependent bounds for arbitrary $N\in \mathbb N$.
- Let $f\in \mathcal C_{{\text {b}}}^{N}(D,\mathbb R^d)$ for some open set $D \subset \mathbb R^d$ and $N \in \mathbb N$. If $\Omega$ is unbounded assume $f(0)=0$. Let $j \in \mathbb N$ be such that $j> n/2$. Let $\mathcal D$ be an $\mathcal H_j$ bounded subset of
  $$\begin{aligned} \{ u \in \mathcal H_j(\Omega ;\mathbb R),~ u(\Omega ) \subset D \} \end{aligned}$$
  and for $R>0$, $k\in \mathbb N$ with $k\ge j$ let
  2.18 $$\begin{aligned} \mathcal D_k = \mathcal D\cap \mathcal B^R_{\mathcal H_k}(0). \end{aligned}$$
  Here $$\mathcal H_k =\mathcal H_{k}(\Omega ;\mathbb R^d) $$. Then,
  $$\begin{aligned} f \in \mathcal C_{{\text {b}}}^{N-k}(\mathcal D_k; \mathcal H_k), \quad \text{ for }\quad k \in \{j,\ldots , N\} \end{aligned}$$
  with *R*-dependent bounds.
- Let *D*, *f* and *j* be as in b) and let $L> n/2$ be such that $\lfloor L\rfloor \le N$. Then
  $$\begin{aligned} f \in \mathcal C_{{\text {b}}}^{N-\lfloor \ell \rfloor }(\mathcal D_{\ell }; \mathcal H_{\ell -1}) \quad \text{ for } \text{ all }\quad \ell \in [j,L], \end{aligned}$$
  with $\mathcal D_\ell$ defined as in (2.18).

### *Proof*

We restrict to the case $d=1$. A generalization to $d>1$ is straightforward.

To prove a) let $\ell >n/2$. Then there exists a constant $c=c(\ell )$ such that for every $u, v \in \mathcal H_{\ell }(\Omega ;\mathbb C)$ we have $uv \in \mathcal H_{\ell }(\Omega ;\mathbb C)$ with

2.19 $$\begin{aligned} ||uv ||_{\mathcal H_{\ell }}^{} \le c \, ||u ||_{\mathcal H_{\ell }(\Omega ;\mathbb C)}^{} \, ||v ||_{\mathcal H_{\ell }(\Omega ;\mathbb C)}^{}, \end{aligned}$$

see, e.g., [1]. Let *f* be analytic on $\mathcal B_{\mathbb C}^\rho$ and let

2.20 $$\begin{aligned} f(z) = \sum _{n=0}^\infty \, a_n \, z^n \, \end{aligned}$$

be the Taylor series of *f* around 0 for $|z|\le \rho$. Let $g :\mathbb R\rightarrow \mathbb R$ be its majorization

$$\begin{aligned} g(s) = \sum _{n=0}^\infty \, |a_n |\, s^{n}. \end{aligned}$$

By applying the algebra inequality (2.19) to each term of the power series expansion (2.20) of *f*(*u*), we see that the series converges for every $u \in \mathcal H_{\ell }$ provided $\ell > n/2$, and that

2.21 $$\begin{aligned} ||f(u) ||_{\mathcal H_{\ell }}^{} \le c^{-1} \, g \left( c \, ||u ||_{\mathcal H_{\ell }}^{} \right) + |a_0|(\sqrt{|\Omega |} - c^{-1}), \end{aligned}$$

where *c* is as in (2.19), $R\le \rho /c$ and $a_0=0$ if $\Omega$ is unbounded. In other words, *f* is analytic and bounded as function from a ball of radius *R* around 0 in $\mathcal H_{\ell }= \mathcal H_{\ell }(\Omega ;\mathbb C)$ to $\mathcal H_{\ell }$. Similarly we see that the same holds for the derivatives of *f*.

To prove b) note that $\mathcal D$ is well-defined because by the Sobolev embedding theorem $\mathcal H_j(\Omega ;\mathbb R) \subseteq \mathcal C_{{\text {b}}}(\Omega ;\mathbb R)$. In [15, Theorem 2.12], the statement was proved in the case $n=1$. The extension to the case $n>1$ is straightforward. Here let us just illustrate the idea of the proof for the example $n=1$, $N=1$ and $j=k=1$. Then $f \in \mathcal C_{{\text {b}}}^1(\mathcal D_1;\mathcal L_2)$ by the Sobolev embedding theorem, but also $f \in \mathcal C_{{\text {b}}}(\mathcal D_1;\mathcal H_1)$ since for this we only need that $\partial _x f(u) = f'(u) \partial _x u \in \mathcal L_2$ with uniform bound in $u \in \mathcal D_1$ which is again true by the Sobolev embedding theorem.

To prove c) note that for $\ell \in [j,L]$ we know from b) that $f \in \mathcal C^{N-\lfloor \ell \rfloor }(\mathcal D_{\lfloor \ell \rfloor }; \mathcal H_{\lfloor \ell \rfloor })$. Since $\mathcal D_{\ell } \subseteq \mathcal D_{\lfloor \ell \rfloor }$ and $\mathcal H_{\lfloor \ell \rfloor } \subseteq \mathcal H_{ \ell -1}$ this implies $f \in \mathcal C^{N-\lfloor \ell \rfloor }(\mathcal D_{\ell }; \mathcal H_{\ell -1})$. $\square$

## Runge–Kutta time semidiscretizations

In this section we apply an A-stable Runge–Kutta method in time to the evolution equation (1.1), and establish well-posedness and regularity of the numerical method on the infinite dimensional space $\mathcal Y$.

Given an (*s*, *s*) matrix $\mathsf a$, and a vector $\mathsf b\in \mathbb R^s$, we define the corresponding Runge–Kutta method by

3.1a $$\begin{aligned}&W = U^0\mathbbm {1} + h\mathsf a(AW+B(W)),\end{aligned}$$

3.1b $$\begin{aligned}&\Psi ^h(U^0) = U^0+h\mathsf b^T(AW+B(W)), \end{aligned}$$

where

$$\begin{aligned} U \mathbbm {1} = \begin{pmatrix} U \\ \vdots \\ U \end{pmatrix}\in \mathcal Y^s\quad \text{ for } \quad U \in \mathcal Y,\quad W = \begin{pmatrix} W^1 \\ \vdots \\ W^s \end{pmatrix},\quad B(W) = \begin{pmatrix} B(W^1) \\ \vdots \\ B(W^s) \end{pmatrix}. \end{aligned}$$

Here, $W^1,\ldots ,W^s$ are the stages of the method, we understand *A* to act diagonally on the vector *W*, i.e., $(AW)^i=AW^i$, and

$$\begin{aligned} (\mathsf aW)^i=\sum _{j=1}^s\mathsf a_{ij}W^j,\quad \mathsf b^TW = \sum _{i=1}^s \mathsf b_iW^i. \end{aligned}$$

We define

$$\begin{aligned} \Vert W\Vert _{\mathcal Y_\ell ^s} := \max _{j=1,\ldots ,s} \Vert W^i\Vert _{\mathcal Y_\ell } \end{aligned}$$

and re-write (3.1a) as

3.2 $$\begin{aligned} W = ({\text {id}}- h\mathsf aA)^{-1}(\mathbbm {1} U^0 + h\mathsf aB(W)), \end{aligned}$$

and (3.1b) as

3.3 $$\begin{aligned} \Psi (U,h)=\Psi ^h(U) = \mathsf S(hA)U+h\mathsf b^T({\text {id}}-h\mathsf aA)^{-1}B(W(U,h)), \end{aligned}$$

where $\mathsf S$ is the stability function, given by

3.4 $$\begin{aligned} \mathsf S(z) = 1 + z\mathsf b^T({\text {id}}-z\mathsf a)^{-1}\mathbbm {1}. \end{aligned}$$

In the following $\mathbb C^-_0=\{z\in \mathbb C:\mathfrak {Re}(z)\le 0\}$. We assume *A*-stability of the numerical method as follows (cf. [12]):

1. $\mathsf S(z)$ from (3.4) is bounded with $|\mathsf S(z)|\le 1$ for all $z\in \mathbb C^-_0$.
2. $\mathsf a$ is invertible and the matrices ${\text {id}}-z\mathsf a$ are invertible for all $z\in \mathbb C^-_0$.

### *Example 3.1*

Gauss–Legendre collocation methods such the implicit midpoint rule satisfy (RK1) and (RK2) [15, Lemma 3.6].

The following result is needed later on, see also [15, Lemmas 3.10, 3.11, 3.13]:

### **Lemma 3.2**

Under assumptions (A), (RK1) and (RK2) there are $h_*>0$, $\Lambda >0$ and $\sigma >0$ such that for $h\in [0,h_*]$

3.5a $$\begin{aligned} ||\mathsf S(hA) ||_{\mathcal Y\rightarrow \mathcal Y}^{}\le 1+\sigma h \end{aligned}$$

3.5b $$\begin{aligned} ||({\text {id}}-h\mathsf aA)^{-1} ||_{\mathcal Y^s\rightarrow \mathcal Y^s}^{}\le \Lambda . \end{aligned}$$

Moreover, for any $k\in \mathbb N_0$, $U \in \mathcal Y_k$, $W \in \mathcal Y_k^s$,

$$\begin{aligned} h\mapsto \mathsf S(hA)U \in \mathcal C_{{\text {b}}}^k([0,h_*]; \mathcal Y), \end{aligned}$$

and

$$\begin{aligned} h \mapsto ({\text {id}}-h\mathsf aA)^{-1}W \!\in \! \mathcal C_{{\text {b}}}^k([0,h_*]; \mathcal Y^s), \quad h\mapsto h({\text {id}}-h\mathsf aA)^{-1}W\!\in \! \mathcal C_{{\text {b}}}^{k+1}([0,h_*]; \mathcal Y^s). \end{aligned}$$

Finally there are $c_{\mathsf S,k} >0$ with

3.5c $$\begin{aligned} \sup _{h \in [0,h_*]} \Vert \partial _h^k \mathsf S(hA) \Vert _{\mathcal Y_k\rightarrow \mathcal Y} \le c_{\mathsf S,k}, \end{aligned}$$

and, with $\Lambda _k:=k! \Vert \mathsf a\Vert ^{k} \Lambda ^{k+1}$, we have for $k\in \mathbb N_0$,

3.5d $$\begin{aligned} \Vert \partial ^k_h( ({\text {id}}-h\mathsf aA)^{-1} ) \Vert _{\mathcal Y^s_{k} \rightarrow \mathcal Y^s} \le \Lambda _k, \quad \Vert \partial ^k_h( h({\text {id}}-h\mathsf aA)^{-1} ) \Vert _{\mathcal Y^s_{k-1} \rightarrow \mathcal Y^s} \le \Lambda _k/\Vert \mathsf a\Vert . \end{aligned}$$

### *Proof*

Most of the statements follow directly from [15, Lemmas 3.10, 3.11, 3.13]. (3.5d) follows from

$$\begin{aligned} \partial _h^k({\text {id}}- h \mathsf aA)^{-1} = k! \, (\mathsf aA)^k\, ({\text {id}}- h \mathsf aA)^{-k-1}. \end{aligned}$$

and

$$\begin{aligned} \partial _h^k [ h ({\text {id}}- h \mathsf aA)^{-1}] = \partial _h^{k-1} ({\text {id}}- h \mathsf aA)^{-2} = k! \, (\mathsf aA)^{k-1} \, ({\text {id}}- h \mathsf aA)^{-k-1}, \end{aligned}$$

see [15, Lemma 3.10]. $$\square $$

Analogously to Theorem 2.2, we require a well-posedness and regularity result for the stage vectors $W^i$, $i=1,\ldots , s$, and the numerical method $\Psi ^h$. The following result is an extension of [15, Theorem 3.14] to non-integer values of $\ell$.

### **Theorem 3.3**

(Regularity of numerical method) Assume that the semilinear evolution equation (1.1) satisfies (A) and (B), and apply a Runge–Kutta method subject to conditions (RK1) and (RK2). Let $R>0$. Then there is $h_*>0$ such that there exist a stage vector *W* and numerical method $\Psi$ which satisfy

3.6a $$\begin{aligned} W^i(\cdot ,h), \Psi (\cdot ,h) \in \mathcal C_{{\text {b}}}^{N} (\mathcal B_0^r;\mathcal B^R_0) \end{aligned}$$

for $$i=1,\ldots , s$$, where

3.6b $$\begin{aligned} r= r(R) = \frac{R}{2 \Lambda }. \end{aligned}$$

with uniform bounds in $h \in [0,h_*]$.

Furthermore, for $\ell \in I^-$, $k\in \mathbb N_0$, $k \le \ell$, we have for $i=1,\ldots , s$,

3.6c $$\begin{aligned} W^i(U,\cdot ), \Psi (U,\cdot ) \in \mathcal C_{{\text {b}}}^k( [0,h_*];\mathcal B_0^R) \end{aligned}$$

with uniform bounds in $U \in \mathcal B^r_\ell$. The bounds on $h_*$, $\Psi$ and *W* depend only on *R*, (3.5), those afforded by assumption (B) on balls of radius *R* and on $\mathsf a$, $\mathsf b$ as specified by the numerical method.

### *Proof*

As in [15] we compute *W* as fixed point of the map $\Pi :\mathcal B^R_{\mathcal Y^s}(0) \times \mathcal B^r_\mathcal Y(0) \times [0,h_*]\rightarrow \mathcal Y^s$, given by

3.7 $$\begin{aligned} \Pi (W,U,h) = ({\text {id}}-h \mathsf aA)^{-1} \mathbbm {1} U + h \mathsf a(1-h \mathsf aA)^{-1} B(W), \end{aligned}$$

using (3.2). To be able to apply the contraction mapping theorem we need to check that $\Pi (W,U,h) \in \mathcal B^{R}_{\mathcal Y^s}(0)$ for $U \in \mathcal B^{r}_\mathcal Y(0)$. For such *U* we have

3.8 $$\begin{aligned} \Vert \Pi (W,U,h) \Vert _{\mathcal Y^s}&\le \Vert ({\text {id}}-h \mathsf aA)^{-1}\mathbbm {1} U\Vert _{\mathcal Y^s} + h ||\mathsf a ||_{}^{}\Lambda M \nonumber \\&\le \Lambda r + h ||\mathsf a ||_{}^{}\Lambda M \le R/2 + h \Lambda ||\mathsf a ||_{}^{}M \le R \end{aligned}$$

for $h\in [0,h_*]$ and $h_*$ small enough, with $M = M_0[R]$. So $\Pi$ maps $\mathcal B^{R}_{\mathcal Y^s}(0)$ to itself. Furthermore there is some $c \in [0,1)$ such that $\Vert \mathrm D\Pi (W,U,h)\Vert _{\mathcal Y^s \rightarrow \mathcal Y^s} \le c$ for $W\in \mathcal B^R_{\mathcal Y^s}(0)$, $W\in \mathcal B^r_\mathcal Y(0)$, $h\in [0,h_*]$ if $h_*$ is small enough, and so $\Pi$ is a contraction. Hence, $W\in \mathcal C_{{\text {b}}}(\mathcal B^{r}_{\mathcal Y}(0)) \times [0,h_*];\mathcal B^{R}_{\mathcal Y^s}(0))$ with *N* derivatives in *U*.

This proves statements (3.6a) and also (3.6c) in the case $k=0$ for *W*. Due to (3.3), these statements also hold true for $\Psi$. In the case $k\ne 0$ it follows from the that $\ell \in I^-$ that the above argument also holds on $\mathcal Y_{\ell -j}$, $j=0,\ldots , k$. Hence there is some $h_*>0$ such that $W^i, \Psi \in \mathcal C_{{\text {b}}}(\mathcal B^r_{\ell -j}\times [0,h_*];\mathcal B^R_{\ell -j})$, $j=0,\ldots , k$, $i=1,\ldots , s$. As shown in [15] for $U \in \mathcal B^r_{\ell }$ the *h* derivatives up to order *k* can then be obtained by implicit differentiation of $\Pi (W,U,h)=W(U,h)$ with $\Pi$ defined above and by differentiating (3.3), cf. the proof of Theorem 2.2. This then implies (3.6c). $\square$

A discretization $y^{n+1}=\psi ^h(y^n)$ of an ordinary differential equation (ODE) $\frac{\mathrm dy}{\mathrm dt}= f(y)$ is said to be of classical order *p* if the local error, i.e., the one-step error, of the numerical method is given by the Taylor remainder of order $p+1$,

3.9 $$\begin{aligned} y(h)-\psi ^h(y^0) = \int _0^h\frac{(h-\tau )^p}{p!}\partial _\tau ^{p+1}(y(\tau )-\psi ^\tau (y^0))\mathrm d\tau . \end{aligned}$$

When considering the local error of a semidiscretization of a PDE on a Hilbert space $\mathcal Y$, the derivatives of the semiflow and numerical method in time and step size respectively are not necessarily defined on the whole space $\mathcal Y$. To obtain global error estimates for semidiscretizations of PDE problems analogous to the familiar results for ODEs, we must consider the local error as a map $\mathcal Z\rightarrow \mathcal Y$, where $\mathcal Z$ is a space of higher regularity. Using the regularity results for the semiflow and its discretization in time, Theorems 2.2 and 3.3, the following can be shown (see [15, Theorem 3.20]): if (A), (B), (RK1) and (RK2) hold, and (in our notation) $\ell \in I^-$, $\ell \ge p+1$ then for fixed $T>0$, $R>0$ there exist constants $c_1,c_2,h_*>0$ such that for every solution $\Phi ^t(U^0)$, $t\in [0,T]$ with $\Vert \Phi ^t(U^0)\Vert _{\mathcal Y_{p+1}} \le R$ and every $h\in [0,h_*]$, we have

3.10 $$\begin{aligned} ||\Phi ^{nh}(U^0)-(\Psi ^h)^n(U^0) ||_{\mathcal Y}^{}\le c_1e^{c_2nh}h^p, \end{aligned}$$

provided that $nh\le T$. In this paper we study the case where the solution *U*(*t*) satisfies $U(t) \in \mathcal Y_\ell$ with $\ell <p+1$, by means of Galerkin truncation.

## Spectral Galerkin truncations

In this section we consider the stability of the semiflow $\Phi ^t$ of (1.1), and the numerical method $\Psi ^h$ defined by (3.1) under truncation to a Galerkin subspace of $\mathcal Y$. As before for $m>0$ we denote by $\mathbb P_m$ the spectral projection operator of *A* on to the set ${\text {spec}}\ (A)\cap \mathcal B^{m}_{\mathbb C}(0)$, and set $\mathbb Q_m={\text {id}}-\mathbb P_m$. In this setting we define $B_m(u_m)=\mathbb P_mB(u_m)$, and consider the projected semilinear evolution equation

4.1 $$\begin{aligned} \frac{ \mathrm du_m}{\mathrm dt}&= Au_m + B_m(u_m) \end{aligned}$$

with flow map $\phi _m^t(u^0_m)=u_m(t)$ for $u_m(0)=u^0_m \in \mathbb P_m \mathcal Y$. Moreover we define $\Phi ^t_m :=\phi ^t_m\circ \mathbb P_m$. The Galerkin truncated semiflow has the same regularity properties as the full semiflow (see Theorem 2.2) uniformly in *m*.

### **Lemma 4.1**

(Regularity of projected semiflow) Assume (A) and (B) and let $R>0$. Then there is $T_*>0$ such that for $m\ge 0$ there exists a projected semiflow $\Phi _m$ which satisfies

4.2a $$\begin{aligned} \Phi ^t_m\in \mathcal C_{{\text {b}}}^{N}(\mathcal B_0^{R/2};\mathcal B_0^R) \end{aligned}$$

with uniform bounds in $t\in [0,T_*]$ and $m\ge 0$. Moreover if $\ell \in I^-$ and $k\in \mathbb N_0$ satisfies $k \le \ell$, then

4.2b $$\begin{aligned} \Phi _m(U) \in \mathcal C_{{\text {b}}}^k([0,T_*];\mathcal B_0^R) \end{aligned}$$

with uniform bounds in $U \in \mathcal B^{R/2}_\ell$ and $m\ge 0$. The bounds on $T_*$ and $\Phi _m$, depend only on *R*, $\omega$ from (2.1), and those afforded by assumption (B) on balls of radius *R*.

In the case $B\equiv 0$ it is clear that for $U^0 \in \mathcal Y_\ell$ we have the estimate $||\Phi ^t(U^0)-\Phi _m^t(U^0) ||_{\mathcal Y}^{} = \mathcal O(m^{-\ell })$ on any finite interval of existence [0, *T*]. With the presence of a nonlinear perturbation $B\ne 0$ a similar result can be obtained by a Gronwall type argument as shown in the lemma below, which gives an appropriate bound for the error of the semiflow incurred in Galerkin truncation. Note that similar results for mixed higher order derivatives in time and initial value are obtained, for integer $\ell$ in [16, Theorems 2.6 and 2.8].

### **Lemma 4.2**

(Projection error for the semiflow) Assume that the semilinear evolution equation (1.1) satisfies (A) and (B), let $\ell >0$, $T>0$ and $\delta >0$. Then for all $U^0$ with

4.3a $$\begin{aligned} \Vert \Phi ^t(U^0)\Vert _{\mathcal Y_\ell } \le R,\quad t \in [0,T] \end{aligned}$$

there is $m_*\ge 0$ such that for $m\ge m_*$ we have $\Phi _m^t(U^0) \in \mathcal B^{R+\delta }_0$ for $t\in [0,T]$, and

4.3b $$\begin{aligned} \Vert \Phi ^t(U^0)-\Phi ^t_m(U^0) \Vert _\mathcal Y= m^{-\ell }R \mathrm e^{(\omega + M')t}= \mathcal O(m^{-\ell }) \end{aligned}$$

for $$m\ge m_*$$ and $$t\in [0,T]$$, where $$M'=M'_0[R+\delta ]$$. Here $$m_* $$ and the order constant depend only on $$\delta $$, *R*, *T*, (2.1) and the bounds afforded by (B) on balls of radius $$R+\delta $$.

### *Proof*

The statement is shown for integer $\ell$ in [16]. We review the argument, which also works for arbitrary $\ell \in I$. To prove (4.3b) we use the mild formulation (2.9) for $\Phi$ and $\Phi _m$. We find

$$\begin{aligned} ||\Phi ^t(U^0)-\Phi _m^t(U^0) ||_{\mathcal Y}^{}&\le || \mathbb Q_m\Phi ^t(U^0) ||_{\mathcal Y}^{} \\&\quad + || \int _0^te^{(t-\tau )A}(\mathbb P_m B(\Phi ^\tau (U^0))-\mathbb P_mB(\Phi ^\tau _m(U^0)))\mathrm d\tau ||_{\mathcal Y}^{}\\&\le m^{-\ell } R + \int _0^{t} \mathrm e^{\omega (t-\tau )} \Vert B(\Phi ^\tau (U^0))-B(\Phi ^\tau _m(U^0))\Vert _\mathcal Y\mathrm d\tau \\&\le m^{-\ell }R+ M' \int _0^{t} \mathrm e^{\omega (t-\tau )}||\Phi ^\tau (U^0)-\Phi _m^\tau (U^0) ||_{\mathcal Y}^{}\mathrm d\tau , \end{aligned}$$

where $M' = M'_0[R+\delta ]$ a bound of $\mathrm DB$ as map from $\mathcal B_0^{R+\delta }$ to $\mathcal E(\mathcal Y)$, see condition (B), and we choose $m_*>0$ big enough such that

4.4 $$\begin{aligned} ||\Phi ^\tau (U^0)-\Phi _m^\tau (U^0) ||_{\mathcal Y}^{} \le \delta \quad \text{ for }\quad \tau \in [0,T]. \end{aligned}$$

Thus, applying a Gronwall type argument, we obtain (4.3b). $\square$

We also consider an *s*-stage Runge–Kutta method applied to the projected semilinear evolution equation (4.1). We denote by $w_m=w_m(u^0_m,h)$ the stage vector of this map, and by $\psi ^h_m(u_m^0)$ the one-step numerical method applied to the projected system (4.1) and define $W_m=w_m\circ \mathbb P_m$, $\Psi ^h_m=\psi ^h_m\circ \mathbb P_m$. Similar to Lemma 4.1 and Lemma 4.2, we have the following results regarding the existence, regularity and error under truncation for the projected numerical method. Note that similar results have been obtained, for integer $\ell$, and mixed derivatives in [16, Theorems 3.2 and 3.6].

### **Lemma 4.3**

(Regularity of projected numerical method and projection error) Assume that the semilinear evolution equation (1.1) satisfies (A) and (B), and apply a Runge–Kutta method subject to conditions (RK1) and (RK2). Let $$R>0$$. Then there is $$h_*>0 $$ such that for $$m\ge 0$$ there exist a stage vector $$W_m$$ and numerical method $$\Psi _m$$ of the projected system (4.1) which satisfy

4.5a $$\begin{aligned} W^i_m(\cdot ,h), \Psi _m(\cdot ,h) \in \mathcal C_{{\text {b}}}^{N} (\mathcal B^{r}_0;\mathcal B^R_0) \end{aligned}$$

for $i=1,\ldots , s$, where *r* is as in (3.6b), with uniform bounds in $h \in [0,h_*]$, $m\ge 0$. Furthermore, for $\ell \in I^-$, $k\in \mathbb N_0$, $k \le \ell$, we have for $i=1,\ldots , s$,

4.5b $$\begin{aligned} W^i_m(U,\cdot ), \Psi _m(U,\cdot ) \in \mathcal C_{{\text {b}}}^k( [0,h_*];\mathcal B^R_0) \end{aligned}$$

with uniform bounds in $U \in \mathcal B^r_\ell$, $m\ge 0$. Finally, if $\ell \in I$, $\ell >0$, then for $m\ge 0$ we get

4.5c $$\begin{aligned} \sup _{ \begin{array}{c} U\in \mathcal B^r_\ell \\ h\in [0,h_*] \end{array} } ||W(U,h)-W_m(U,h) ||_{\mathcal Y^s}^{}=\mathcal O(m^{-\ell }) \end{aligned}$$

and

4.5d $$\begin{aligned} \sup _{ \begin{array}{c} U\in \mathcal B^r_\ell \\ h\in [0,h_*] \end{array} } ||\Psi (U,h)-\Psi _m(U,h) ||_{\mathcal Y}^{}=\mathcal O(m^{-\ell }). \end{aligned}$$

The bounds on $h_*$, $\Psi _m$ and $W_m$ and the order constants depend only on *R*, (3.5), those afforded by assumption (B) on balls of radius *R* and on $\mathsf a$, $\mathsf b$ as specified by the numerical method.

### *Proof*

The statements (4.5a) and (4.5b) are shown exactly as in the proof of Theorem 3.3 and (4.5c), (4.5d) are shown for integer $\ell$ in [16]. The same arguments are valid for arbitrary $\ell \in I$ as well, we review the proof for completeness. From the formulation (3.2) of the stage vectors $W^i,W^i_m$, $i=1,\ldots ,s$, we find

4.6 $$\begin{aligned} ||W(U,h)-W_m(U,h) ||_{\mathcal Y^s}^{}&\le ||({\text {id}}-h\mathsf aA)^{-1} ||_{\mathcal Y^s\rightarrow \mathcal Y^s}^{}||\mathbb Q_mU ||_{\mathcal Y}^{} \nonumber \\&\quad + ||h \mathsf a({\text {id}}-h\mathsf aA)^{-1} \mathbb Q_mB(W) ||_{\mathcal Y^s}^{} \nonumber \\&\quad +h||({\text {id}}-h\mathsf aA)^{-1} ||_{\mathcal Y^s\rightarrow \mathcal Y^s}^{}||\mathsf a ||_{}^{}||\mathbb P_m(B(W)-B(W_m))) ||_{\mathcal Y^s}^{} \nonumber \\&\le \Lambda ||\mathbb Q_m U ||_{\mathcal Y}^{} + h \Vert \mathsf a\Vert \Lambda m^{-\ell }M_\ell [R] \nonumber \\&\quad + h \Lambda ||\mathsf a ||_{}^{} ||\mathbb P_m B(W(U,h))-\mathbb P_m B(W_m(U,h)) ||_{\mathcal Y^s}^{} \nonumber \\&\le \Lambda ||U ||_{\mathcal Y_\ell }^{}m^{-\ell }+ h \Vert \mathsf a\Vert \Lambda m^{-\ell }M_\ell [R] \nonumber \\&\quad + h \Lambda ||\mathsf a ||_{}^{} M' ||W(U,h)-W_m(U,h) ||_{\mathcal Y^s}^{} \end{aligned}$$

with an order constant uniform in $U\in \mathcal B^r_{\ell }$. Here $M' = M'_0[R]$ and we used (3.5b) and (2.4). Solving for $||W(U,h)-W_m(U,h) ||_{\mathcal Y^s}^{}$ and taking the supremum over $h\in [0,h_*]$ and $U \in \mathcal B^r_{\ell }$ we get (4.5c).

Similarly for the numerical method using (3.3), (3.5) and (2.4) we estimate

4.7 $$\begin{aligned} ||\Psi ^h(U)-\Psi ^h_m(U) ||_{\mathcal Y}^{}&\le ||\mathsf S(hA) ||_{\mathcal Y\rightarrow \mathcal Y}^{}||\mathbb Q_mU ||_{\mathcal Y}^{} + \Vert \mathbb Q_m \mathsf bh({\text {id}}- h \mathsf aA)^{-1} B(W)\Vert _{\mathcal Y^s} \nonumber \\&\quad + h \Vert \mathsf b\Vert \Lambda ||\mathbb P_m(B(W)- B(W_m)) ||_{\mathcal Y^s}^{} \nonumber \\&\le (1+\sigma h)||U ||_{\mathcal Y_\ell }^{}m^{-\ell } + s\Vert \mathsf b\Vert h \Lambda m^{-\ell } M_\ell [R] \nonumber \\&\quad + s h||\mathsf b ||_{}^{}\Lambda M'||W(U)-W_m(U) ||_{\mathcal Y^s}^{} \nonumber \\&\le (1+\sigma h)||U ||_{\mathcal Y_\ell }^{}m^{-\ell } +s \Vert \mathsf b\Vert h \Lambda m^{-\ell } M_\ell [R] \nonumber \\&\quad + s h||\mathsf b ||_{}^{}\Lambda M' \mathcal O(m^{-\ell }). \end{aligned}$$

Here we used (4.5c) in the last line. $\square$

## Trajectory error bounds for non-smooth data

In this section we consider the convergence of the global error

5.1 $$\begin{aligned} E^n(U,h)=||\Phi ^{nh}(U)-(\Psi ^h)^n(U) ||_{\mathcal Y}^{} \end{aligned}$$

as $h\rightarrow 0$ for non-smooth initial data. As mentioned above, cf. (3.10), [15, Theorem 3.20] states that we have $E^n(U^0,h)=\mathcal O(h^p)$ in some interval [0, *T*], $0\le nh\le T$, given sufficient regularity of the semiflow and time semidiscretization to bound the local error given by the Taylor expansion to order $p+1$ as a map

5.2 $$\begin{aligned} U\mapsto \left||\int _0^h\frac{(h-\tau )^p}{p!}\partial _\tau ^{p+1}(\Phi ^\tau (U^0)-\Psi ^\tau (U^0))\ d\tau \right||_{\mathcal Y}^{}, \end{aligned}$$

see (3.9). As stated by Theorems 2.2 and 3.3, this is the case provided $\ell \in I^-$, $\ell \ge p+1$. In this paper we study the order $q=q(\ell )$ of convergence of the global error for non-smooth initial data $U^0 \in \mathcal Y_\ell$, $\ell \in I^-$, $\ell <p+1$, such that $E^n(U,h)=\mathcal O(h^q)$ and show that we obtain $q(\ell ) = p\ell /(\ell +1)$ as Brenner and Thomée [3] and Kovács [9] did for linear strongly continuous semigroups.

The implicit midpoint rule, the simplest Gauss–Legendre method, satisfies the conditions (RK1) and (RK2), see Example 3.1 with $p=2$. Figure 1 shows the order of convergence of the implicit midpoint rule applied to the semilinear wave equation (2.11) with $V'(u)=u-4u^2$ for $\ell =j/2$, $j=0,\ldots , 6$, on the integration interval $t \in [0,0.5]$, using a fine spatial mesh (we use $N=1000$ grid points on $[0, 2\pi ]$). As initial values we choose $U^0= (u^0, v^0) \in \mathcal Y_\ell$ where

$$\begin{aligned} u^0(x) = \sum _{k=0}^{N=1} \frac{c_u}{k^{\ell + 1/2+ \epsilon }} (\cos kx + \sin kx), \quad v^0(x) = \sum _{k=0}^{N=1} \frac{c_v}{k^{\ell + 1/2+ \epsilon }} (\cos kx + \sin kx). \end{aligned}$$

Here $c_u$ and $c_v$ are such that $\Vert U^0\Vert _{\mathcal Y_\ell }= 1$, with $U^0 = (u^0, v^0)$, and $\epsilon = 10^{-8}$. From Theorem 2.2, with $\mathcal Y$ replaced by $\mathcal Y_\ell$, we know that there is some $T_*>0$ such that $\Phi ^t(U^0) \in \mathcal B^R_\ell$ for $U^0 \in \mathcal Y_\ell$ so that the assumption (5.20) of our convergence result, Theorem 5.3 below, is satisfied. We integrate the semilinear wave equation with the above initial data for the time steps $h = 0.1, 0.095, 0.09, 0.085,\ldots , 0.05$, when $\ell >0$. At $\ell =0$, to reduce computational effort, we only used the time steps $h = 0.1, 0.09, \ldots 0.05$. To estimate the trajectory error, we compare the numerical solution to a solution calculated using a much smaller time step, $\tilde{h} = 10^{-3}$ for $\ell >0$ and $\tilde{h} = 10^{-4}$ for $\ell =0$. From the assumption $E_n(h) = c h^{q}$ we get $\log E_n(h) = \log c + q \log h$. Fitting a line to those data, we take the gradient of the line as our estimated order of convergence of the trajectory error. The decay in $q(\ell )$ as $\ell$ decreases from 3 is clearly shown. Note that the order of convergence does not decrease to exactly 0 at $\ell =0$ and is slightly better than predicted by our theory when $\ell =2.5$. This is because we simulate a space-time discretization rather than a time semidiscretization. Moreover at $\ell =0$, despite the fact that we already use a finer time step size, the approximation of the exact solution is not that accurate as the order of convergence for the time-semidiscretization vanishes at $\ell =0$.

In the rest of this section, equipped with the results of Sect. 4 on the stability of the semiflow and the numerical method under Galerkin, truncation we estimate the growth with *m* of the local error of a Runge–Kutta method (3.1), subject to (RK1) and (RK2), applied to the projected equation (4.1) subject to (A) and (B) for non-smooth initial data. In this setting, by coupling *m* and *h* and balancing the projection error and trajectory error of the projected system, we obtain an estimate for $q(\ell )$ that describes the convergence of the numerical method for the semilinear evolution equation (1.1) as observed in Fig. 1, see Sect. 5.2.

### Preliminaries

We start with some preliminary lemmas.

#### **Lemma 5.1**

(*m*-dependent bounds for derivatives of $\Phi _m$) Assume that the semilinear evolution equation (1.1) satisfies (A) and (B) and choose $\ell \in I^-$, $T>0$, $m_*\ge 0$ and $R>0$. Then for all $U^0$ with

5.3a $$\begin{aligned} \Phi ^t_m(U^0) \in \mathcal B_\ell ^R\quad \text{ for }\quad t\in [0,T],\quad m\ge m_*, \end{aligned}$$

and for all $k\in \mathbb N_0$, $k\le \ell$ we have

5.3b $$\begin{aligned} \Phi _m(U^0) \in \mathcal C_b^{k}( [0,T];\mathcal B_0^R) \end{aligned}$$

with bounds uniform in $U^0$ and $m\ge m_*$. Further, choose $k\in \mathbb N_0$ with $\ell \le k \le N$. Then for all $U^0$ satisfying (5.3a), (5.3b) still holds, but with *m*-dependent bounds which are uniform in $U^0$. Moreover for all such $U^0$, $\ell \le k \le N$,

5.3c $$\begin{aligned} ||\partial _t^k\Phi ^t_m(U^0) ||_{\mathcal C_{{\text {b}}}([0,T];\mathcal Y)}^{}= \mathcal O(m^{k-\ell }), \end{aligned}$$

with bounds uniform in $U^0$. The bounds and order constants only depend on *T*, *R*, (2.1) and the bounds from assumption (B).

#### *Proof*

Due to Lemma 4.1 statement (5.3b) is non-trivial only if $\ell \ge 1$. In this case let $u_m(t) = \Phi _m^t(U^0)$. From Lemma 4.1 with $\mathcal Y$ replaced by $\mathcal Y_\ell$, using (5.3b) we also get $u_m \in \mathcal C_{{\text {b}}}([0,T];{\mathcal B^R_{\ell }})$. From (5.3a) and (4.1) we conclude that $\partial _t u_m \in \mathcal C_{{\text {b}}}([0,T];{\mathcal Y_{\ell -1}})$ and thus, $u_m \in \mathcal C_{{\text {b}}}([0,T];{\mathcal Y_\ell }) \cap \mathcal C_{{\text {b}}}^1([0,T];{\mathcal B_{\ell -1}^R})$ with bounds uniform in $m\ge m_*$ and $U^0$ satisfying (5.3a). That proves (5.3b) for $k=1$. If $\ell \ge 2$ then from (4.1) we get $\partial _t u_m \in \mathcal C_{{\text {b}}}^1([0,T];{\mathcal Y_{\ell -2}})$ and therefore $u_m \in \mathcal C_{{\text {b}}}^2([0,T];{\mathcal B_{\ell -2}^R})$. Inductively this proves that

5.4 $$\begin{aligned} \Phi _m(U^0)\in \mathcal C_{{\text {b}}}^{k}([0,T]; \mathcal B_{\ell -k}^R) \end{aligned}$$

for $k\le \ell$ with uniform bounds in $m\ge m_*$ and in all $U^0$ satisfying (5.3a). This proves (5.3b) for $k \le \ell$ with *m* independent bounds.

To prove (5.3c) we proceed by induction over $k = \lceil \ell \rceil ,\ldots ,N$.

We consider the cases $\ell <1$ and $\ell \ge 1$ separately. If $\ell <1$ then from (4.1) we have

$$\begin{aligned} \Vert \partial _t u_m\Vert _{\mathcal C([0,T];\mathcal Y)} \!\le \! \Vert A_m u_m\Vert _{\mathcal C([0,T];\mathcal Y)}\!+\! M \le m^{1-\ell } \Vert u_m\Vert _{\mathcal C([0,T];\mathcal Y_\ell )}+ M =O(m^{1-\ell }) \end{aligned}$$

where $M=M_0[R]$, with order constant independent of $m\ge m_*$ and of $U^0$ satisfying (5.3a). This then immediately shows (5.3c) for $k= \lceil \ell \rceil =1$.

If $\ell \ge 1$, $\ell \in \mathbb Z$ then the start of the induction is $k=\ell$, and the left hand side of (5.3c) is bounded by (5.3b).

If $\ell \ge 1$, $\ell \notin \mathbb Z$ then the start of the induction is $k = \lceil \ell \rceil > \ell$. Using (5.4) we can bound the $\lfloor \ell \rfloor$-th derivative independent of *m* in the $\mathcal Y_{\ell -\lfloor \ell \rfloor }$ norm. Using the Faà di Bruno formula [5] we find that for any $i \in \mathbb N$, $i< N$,

5.5 $$\begin{aligned} \partial _t^{i+1}u_m&= \partial _t^i(Au_m+B_m(u_m))\nonumber \\&= A(\partial _t^iu_m)+ \sum _{1\le \beta \le i}\frac{i! \mathrm D^\beta _uB_m(u_m)}{j_1!\cdots j_i!} \prod _{\alpha =1}^i\left( \frac{\partial _t^\alpha u_m}{\alpha !}\right) ^{j_\alpha }, \end{aligned}$$

where $\beta =j_1+\cdots +j_i$ and the sum is over all $j_\alpha \in N_0$, $\alpha = 1,\ldots , i$, with $j_1+2j_2+\cdots + ij_i=i$. We consider (5.5) with *i* replaced by $\lfloor \ell \rfloor$. Then the second term in the last line of (5.5) is bounded independent of $m\ge m_*$ due to (5.3b). Furthermore, since $\partial _t^{\lfloor \ell \rfloor }u_m\in \mathcal Y_{\ell -\lfloor \ell \rfloor }$ by (5.4) with uniform bound in $m\ge m_*$, we estimate

$$\begin{aligned} \Vert A(\partial _t^{\lfloor \ell \rfloor }u_m)\Vert _\mathcal Y=\Vert A^{1+\lfloor \ell \rfloor -\ell }(A^{\ell -\lfloor \ell \rfloor } \partial _t^{\lfloor \ell \rfloor }u_m)\Vert _\mathcal Y=\mathcal O(m^{1+\lfloor \ell \rfloor -\ell }), \end{aligned}$$

where we have used the first inequality of (2.4). So (5.3c) also holds true for $k =i+1= \lceil \ell \rceil$ when $\ell > 1$, $\ell \notin \mathbb Z$.

Now fix an integer *k* and assume that (5.3c) holds for all integers *i* such that $\ell \le i \le k$. We now use (5.5) with $i=k$ to estimate $\Vert \partial _t^{k+1}u_m\Vert _\mathcal Y$. By the first inequality of (2.4) and the induction hypothesis the first term on the second line of (5.5) is $\mathcal O(m^{k+1-\ell })$. Moreover, by (5.3b) and the induction hypothesis, the $\mathcal Y$ norm of the second term is of order $\mathcal O(m^n)$ with $n=0$ if $j_{\lceil \ell \rceil } + \cdots + j_k=0$ and

$$\begin{aligned} n= (\lceil \ell \rceil -\ell )j_{\lceil \ell \rceil }+\cdots + (k-\ell )j_k\le k-\ell . \end{aligned}$$

if $j_{\lceil \ell \rceil } + \cdots + j_k>0$. Thus we see that the right hand term of (5.5), with $i=k$, is $\mathcal O(m^{k+1-\ell })$ as well. $\square$

#### **Lemma 5.2**

(*m*-dependent bounds for derivatives of $\Psi _m$ and $W_m$) Assume that the semilinear evolution equation (1.1) satisfies (A) and (B), and apply a Runge–Kutta method subject to (RK1) and (RK2). Choose $\ell \in I^-$ and $k\in \mathbb N_0$ with $\ell \le k\le N$. Let $R>0$ and define *r* as in (3.6b). Then there is $h_*>0$ such that for $m\ge 0$ and $i=1,\ldots , s$,

5.6 $$\begin{aligned} W_m^i(U,\cdot ),\Psi _m(U,\cdot ) \in \mathcal C_b^{k}([0,h_*];\mathcal B_0^R) \quad \text{ for } \quad i=1,\ldots ,s \end{aligned}$$

with *m*-dependent bounds which are uniform in $U \in \mathcal B_\ell ^r$. Moreover

5.7 $$\begin{aligned} \sup _{ \begin{array}{c} U\in \mathcal B_\ell ^r \\ h\in [0,h_*] \end{array}} ||\partial _h^{k}\Psi ^h_m(U) ||_{\mathcal Y}^{} = \mathcal O(m^{k-\ell }), \quad \sup _{ \begin{array}{c} U\in \mathcal B_\ell ^r\\ h\in [0,h_*] \end{array} }||\partial _h^{k}W_m(U,h) ||_{\mathcal Y^s}^{} = \mathcal O(m^{k-\ell }). \end{aligned}$$

The order constants in (5.7) depend only *R*, (3.5), $\mathsf a$ and $\mathsf b$ from the numerical method and the bounds afforded by (B) on balls of radius *R*.

#### *Proof*

By Lemma 4.3, with $\mathcal Y$ replaced by $\mathcal Y_{\ell -j}$,

5.8 $$\begin{aligned} \Psi _m, W_m^i \in \mathcal C_b^{j} ([0,h_*]; \mathcal B^R_{\ell -j}), \end{aligned}$$

for $i=1,\ldots , s$, $j=1\ldots , \lfloor \ell \rfloor$, with bounds independent over $m\ge 0$ and $U \in \mathcal B^r_\ell$. From (3.3) we formally obtain

5.9 $$\begin{aligned} \partial _h^k\Psi _m^h(U,h) \!=\! \partial ^k_h\mathsf S(hA)\mathbb P_m U+ \sum _{j=0}^k {k \atopwithdelims ()j} \mathsf b^T \partial ^{k-j}_h ( h({\text {id}}-h\mathsf aA)^{-1}) \partial _h^j \mathbb P_m B(W_m(U,h)). \end{aligned}$$

By (3.5c) and (2.4) there are $h_*>0$, $c_{\mathsf S,k}$ such that for all $h\in [0,h_*]$ and $k\ge \ell$

5.10 $$\begin{aligned} \Vert \partial ^k_h\mathsf S(hA)\mathbb P_m \Vert _{\mathcal Y_\ell \rightarrow \mathcal Y} \le \Vert \partial ^k_h\mathsf S(hA) \Vert _{\mathcal Y_k \rightarrow \mathcal Y} \Vert \mathbb P_m \Vert _{\mathcal Y_\ell \rightarrow \mathcal Y_k} \le c_{\mathsf S,k} m^{k-\ell }. \end{aligned}$$

In addition (3.5d) shows that for $n\in \mathbb N$ with $n-1 \ge \ell$

5.11 $$\begin{aligned} \Vert \partial ^n_h( h({\text {id}}-h\mathsf aA)^{-1} \mathbb P_m)\Vert _{\mathcal Y^s_\ell \rightarrow \mathcal Y^s}&\le \Vert \partial ^n_h( h({\text {id}}-h\mathsf aA)^{-1} )\Vert _{\mathcal Y^s_{n-1} \rightarrow \mathcal Y^s} \Vert \mathbb P_m\Vert _{\mathcal Y^s_\ell \rightarrow \mathcal Y^s_{n-1}}\nonumber \\&\le \frac{\Lambda _{n}}{\Vert \mathsf a\Vert } m^{n-1-\ell }. \end{aligned}$$

Using (5.11) (with $\ell$ replaced by $\ell -j$ and *n* by $k-j$) and (5.8), we can estimate the *j*-th term in the sum of (5.9) for $0\le j\le \ell \le k$ as follows:

5.12 $$\begin{aligned} \Vert&\partial ^{k-j}_h ( h({\text {id}}-h\mathsf aA)^{-1}) \partial _h^j \mathbb P_m B(W_m(U,h))\Vert _{\mathcal Y^s}\nonumber \\&\le \Vert \partial ^{k-j}_h ( h({\text {id}}-h\mathsf aA)^{-1})\mathbb P_m \Vert _{ \mathcal Y^s_{\ell -j}\rightarrow \mathcal Y^s} \Vert \partial _h^j B(W_m(U,h))\Vert _{\mathcal Y_{\ell -j}^s} \nonumber \\&\le O(m^{ k-\ell }). \end{aligned}$$

To obtain the first estimate of (5.7) assume that there is $b_j>0$ such that

5.13 $$\begin{aligned} \Vert \partial _h^j \mathbb P_m B(W_m(U,h))\Vert _\mathcal Y\le b_j m^{j-\ell } \end{aligned}$$

for all $h\in [0,h_*]$, $U \in \mathcal B^r_\ell$ and $k\ge j \ge \ell$. This will be proved below. Then, using (5.11) and (5.13) we can estimate the *j*-th term in the sum of (5.9) for $j\ge \ell$ as follows:

5.14 $$\begin{aligned}&\Vert \partial ^{k-j}_h ( h({\text {id}}-h\mathsf aA)^{-1}) \partial _h^j \mathbb P_m B(W_m(U,h))\Vert _{\mathcal Y^s}\nonumber \\&\quad \le \Vert \partial ^{k-j}_h ( h({\text {id}}-h\mathsf aA)^{-1})\mathbb P_m \Vert _{ \mathcal Y^s\rightarrow \mathcal Y^s} \Vert \partial _h^j B(W_m(U,h))\Vert _{\mathcal Y^s}\nonumber \\&\quad \le \frac{\Lambda _{k-j}}{\Vert \mathsf a\Vert } m^{k-j} b_j m^{j-\ell } = O(m^{k-\ell }). \end{aligned}$$

These estimates, with (5.9) and (5.10), then prove the first estimate of (5.7).

To prove (5.13) and the second estimate of (5.7), differentiate (3.2) *k* times in *h*:

5.15 $$\begin{aligned} \partial _h^k W_m = \partial _h^{k}({\text {id}}- h\mathsf aA)^{-1}\mathbbm {1}\mathbb P_m U + \sum _{j=0}^k{k \atopwithdelims ()j}\partial _h^{k-j}(h\mathsf a({\text {id}}- h\mathsf aA)^{-1}\mathbb P_m) \partial _h^j B(W_m). \end{aligned}$$

By (3.5d) and (2.4), for $k\ge \ell$,

5.16 $$\begin{aligned} \sup _{h\in [0,h_*]} \Vert \partial _h^k({\text {id}}- h\mathsf aA)^{-1}\mathbbm {1}\mathbb P_m \Vert _{\mathcal Y_\ell ^s\rightarrow \mathcal Y^s} \le \Lambda _k m^{k-\ell }. \end{aligned}$$

Now we show inductively the second estimate of (5.7) and estimate (5.13) for $k = \lceil \ell \rceil ,\ldots ,N$. If $\ell \in \mathbb N_0$ then the start of the induction is $k = \ell$, and the required estimates are given by Theorem 4.3. If $\ell \notin \mathbb N_0$, then the start of the induction is $k = \lceil \ell \rceil > \ell$. If $k= \lceil \ell \rceil$ then, due to (5.16), the first term in (5.15) is of order $\mathcal O(m^{k-\ell })$, and all other terms in the sum of (5.15) are bounded due to (3.5d) and (5.8) except when $j=k$ in the sum. Hence, using (3.5b),

5.17 $$\begin{aligned} \sup _{\begin{array}{c} h\in [0,h_*]\\ U \in \mathcal B^r_\ell \end{array}} \Vert \partial _h^k W_m(U,h)\Vert _{\mathcal Y^s} \le \mathcal O(m^{k-\ell }) + \Lambda \Vert \mathsf a\Vert h_* \sup _{\begin{array}{c} h\in [0,h_*]\\ U \in \mathcal B^r_\ell \end{array}}\Vert \partial _h^k B(W_m(U,h))\Vert _{\mathcal Y^s}. \end{aligned}$$

Now we use the Faà di Bruno formula (5.5) again:

5.18 $$\begin{aligned} \partial _h^k B(W_m(U,h)) = \sum _{1\le \beta \le k}\frac{k! \mathrm D^\beta _wB_m(W_m(U,h))}{j_1!\cdots j_k!} \prod _{\alpha =1}^k\left( \frac{\partial _h^\alpha W_m(U,h)}{\alpha !}\right) ^{j_\alpha } \end{aligned}$$

where $\beta =j_1+\cdots +j_k$ and the sum is over all $j_\alpha \in N_0$, $\alpha =1,\ldots , k$ with $j_1+2j_2+\cdots kj_k=k$. We see that all terms on the right hand side of (5.18) contain *h*-derivatives of order at most $k-1$ and are therefore bounded and in particular $\mathcal O(m^{k-\ell })$, except when $\beta =j_k=1$ and $j_\alpha =0$ for $\alpha \ne k$. So we obtain

5.19 $$\begin{aligned} \sup _{\begin{array}{c} h\in [0,h_*]\\ U \in \mathcal B^r_\ell \end{array}} \Vert \partial _h^k B(W_m(U,h))\Vert _{\mathcal Y^s}&\le \mathcal O(m^{k-\ell }) + \sup _{\begin{array}{c} h\in [0,h_*]\\ U \in \mathcal B^r_\ell \end{array}} \Vert \mathrm DB(W_m(U,h)) \partial _h^k W_m(U,h)\Vert _{\mathcal Y^s} \nonumber \\&\le \mathcal O(m^{k-\ell }) + M'_0[R] \sup _{\begin{array}{c} h\in [0,h_*]\\ U \in \mathcal B^r_\ell \end{array}} \Vert \partial _h^k W_m(U,h)\Vert _{\mathcal Y^s}. \end{aligned}$$

Substituting this into (5.17) gives the second estimate of (5.7) for $k = \lceil \ell \rceil$ and $h_*$ small enough. Resubstituting this estimate into (5.19) also shows (5.13) for $k=\lceil \ell \rceil$.

Now assume these estimates hold true for all $\hat{k} \in N_0$ with $\ell \le \hat{k}\le k-1$ and let $k\le N$. Then, using the induction hypothesis and the above estimates, in particular (5.12), (5.13), (5.14) and (5.16), all terms in (5.15) are $\mathcal O(m^{k-\ell })$ except when $j = k$ in the sum. We deduce that (5.17) remains valid under the induction hypothesis. Moreover, by the induction hypothesis, each term in the sum of the Faà di Bruno formula (5.18) with $j_k=0$ is of order $\mathcal O(m^n)$ with $n=0$ if $j_{\lceil \ell \rceil }+ \cdots + j_{k-1}=0$ and

$$\begin{aligned} n = (\lceil \ell \rceil -\ell )j_{\lceil \ell \rceil }+\cdots + (k-1-\ell )j_{k-1}\le k-\ell \end{aligned}$$

if $j_{\lceil \ell \rceil }+ \cdots + j_{k-1}>0.$ Hence (5.19) remains valid, and we deduce (5.13) and the second estimate of (5.7) as before. $\square$

### Trajectory error for nonsmooth data 

Now we are ready to prove our main result:

#### **Theorem 5.3**

(Trajectory error for nonsmooth data) Assume that the semilinear evolution equation (1.1) satisfies (A) and (B) and apply a Runge–Kutta method (3.1) subject to (RK1) and (RK2). Let $$\ell \in I^-$$, $$0< \ell \le p+1 $$, and fix $$T>0$$ and $$R>0$$. Then there exist constants $$h_*>0$$, $$c_1>0$$, $$c_2>0$$ such that for every $$U^0 $$ with

5.20 $$\begin{aligned} \Vert \Phi ^t(U^0)\Vert _{\mathcal Y_\ell } \le R, \quad \text{ for }\quad t\in [0,T] \end{aligned}$$

and for all $h\in [0,h_*]$ we have

5.21 $$\begin{aligned} \Vert \Phi ^{nh}(U^0) -( \Psi ^h)^n(U^0)\Vert _\mathcal Y\le c_1e^{c_2nh}h^{p\ell /(p+1)}, \end{aligned}$$

provided that $nh\le T$. The constants $h_*$, $c_1$ and $c_2$ depend only on *R*, *T*, (2.1), (3.5), $\mathsf a$, $\mathsf b$ from the numerical method and the bounds afforded by (B).

**Figure:**
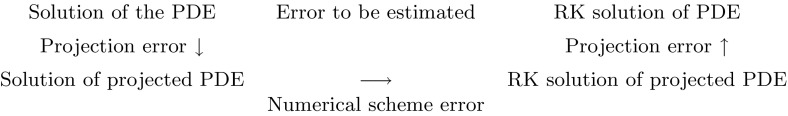


*Proof of Theorem* 5.3. The proof consists of several steps, as outlined in the diagram below:

We want to estimate the error of the Runge Kutta time discretization of the evolution equation (first line of the diagram). To do this, in a first step, we discretize in space by a Galerkin truncation. We estimate the projection error and prove regularity of the solution $u_m(t)$ of the projected system (first column in the diagram). In the second step of the proof we investigate the error of the time discretization of the space-discretized system (third row in the diagram) and couple the spatial discretization parameter *m* with the time step size *h* in suitable way. In the third step of the proof (third column of the diagram) we prove regularity of the space-time discretization and estimate the projection error of the Runge Kutta time discretization. This concludes the proof.

*Step 1 *(*Regularity of solution of the projected system*) In a first step we aim to prove regularity of the continuous solution of the projected system $u_m(t) = \phi _m^t(\mathbb P_m U^0) =\Phi _m^t( U^0)$ which will be needed later. For the proof we denote *R* from (5.20) as $R_\Phi$ to indicate that it is a bound on $\Phi ^t(U^0)$. We will prove that there is some $r_\phi >0$ such that

5.22 $$\begin{aligned} \Vert \phi _m^t(\mathbb P_m U^0) \Vert _{\mathcal Y_\ell } \le r_\phi \end{aligned}$$

uniformly in $U^0$ satisfying (5.20) and $m\ge m_*$, $t\in [0,T]$, where $m_*\ge 0$ is sufficiently large. Fix $\delta >0$. Then we have

5.23 $$\begin{aligned} \Vert \Phi _m^t(U^0)) \Vert _{\mathcal Y_\ell }&\le \Vert \mathbb P_m \Phi ^t(U^0) -\Phi _m^t(U^0) \Vert _{\mathcal Y_\ell } + \Vert \mathbb P_m \Phi ^t(U^0) \Vert _{\mathcal Y_\ell } \nonumber \\&\le m^\ell \Vert \mathbb P_m \Phi ^t(U^0) -\Phi _m^t(U^0) \Vert _{\mathcal Y} + \Vert \Phi ^t(U^0) \Vert _{\mathcal Y_\ell } \nonumber \\&\le R_\Phi \mathrm e^{(\omega + M')t} + R_\Phi = r_\phi \end{aligned}$$

for $U^0$ satisfying (5.20) and $m\ge m_*$. Here $M' = M'_0[R_\Phi + \delta ]$ and we used (2.4) in the second estimate and Lemma 4.2 and (5.20) in the final estimate. This proves (5.22).

*Step 2 *(*Trajectory error of the time discretized projected system*) Next we aim to estimate the trajectory error of the time discretization of the projected system. First note that by Theorem 4.3 (with *r* replaced by $2r_\phi$ and consequently *R* by $4 r_\phi \Lambda$) there is $h_*>0$ such that for $m \ge 0$, $h \in [0,h_*]$ we have $W^i_h, \Psi ^h_m \in \mathcal C_{{\text {b}}}^1(\mathcal B^{2r_\phi }_0;\mathcal Y)$, $i=1,\ldots , s$, with uniform bounds in $m \ge 0$, $h \in [0,h_*]$. Moreover, using (3.3), (3.5a) and (3.5b) we obtain the following bound for $h \in [0,h_*]$ to be used later:

5.24 $$\begin{aligned} \sup _{ U \in \mathcal B^{2r_\phi }_0} \Vert \mathrm D\Psi _m^h(U)\Vert _{ \mathcal Y\rightarrow \mathcal Y}&\le \Vert \mathsf S(hA)\Vert _{ \mathcal Y\rightarrow \mathcal Y} + h\Lambda \Vert \mathsf b\Vert M' \Vert W_m'(U)\Vert _{ \mathcal Y\rightarrow \mathcal Y^s}\nonumber \\&\le 1 + \sigma h + h \Lambda \Vert \mathsf b\Vert M' \Vert W_m'(U)\Vert _{\mathcal Y\rightarrow \mathcal Y^s}=: 1 + \sigma _\Psi h . \end{aligned}$$

where $$M' = M'_0[4 r_\phi \Lambda ]$$.

Now we define the global error of the projected system, for $jh\le T$,

5.25 $$\begin{aligned} E_m^j(U^0,h)=||\Phi ^{jh}_m(U^0)-(\Psi ^h_m)^j(U^0) ||_{\mathcal Y}^{}. \end{aligned}$$

We estimate for any $U^0$ satisfying (5.20) and for all $(n+1)h\le T$, $h\in [0,h_*]$, $m\ge m_*$,

5.26a $$\begin{aligned} E_m^{n+1}(&U^0, h) = ||\Phi ^{(n+1)h}_m( U^0) - (\Psi ^h_m)^{n+1}(U^0) ||_{\mathcal Y}^{} \nonumber \\&\le || \Phi ^h_m(\Phi _m^{nh}(U^0)) \!-\! \Psi ^h_m(\Phi _m^{nh}(U^0)) ||_{\mathcal Y}^{} \!+\! || \Psi ^h_m(\Phi _m^{nh}(U^0)) \!-\! \Psi ^h_m((\Psi ^h_m)^{n}(U^0)) ||_{\mathcal Y}^{} \nonumber \\&\le \frac{h^{p+1}}{(p+1)!}\sup _{\tau \in [0,h]} \left( ||\partial _\tau ^{p+1}\Phi ^\tau _m(\Phi _m^{nh}(U^0)) ||_{\mathcal Y}^{} +||\partial _\tau ^{p+1}\Psi ^\tau _m(\Phi _m^{nh}(U^0))) ||_{\mathcal Y}^{} \right) \nonumber \\&\quad + \sup _{\theta \in [0,1]} ||\mathrm D\Psi ^h_m(\Phi _m^{nh}(U^0)+\theta ((\Psi ^h_m)^{n}(U^0)-\Phi _m^{nh}(U^0))) ||_{\mathcal Y\rightarrow \mathcal Y}^{} \cdot E_m^n(U^0,h) \end{aligned}$$

5.26b $$\begin{aligned}&\le \frac{h^{p+1}}{(p+1)!} \left( ||\partial _t^{p+1}\Phi _m^t(U^0) ||_{\mathcal Y}^{}+\sup _{t\in [0,T]}\sup _{h\in [0,h_*]} ||\partial _h^{p+1}\Psi ^h_m(\Phi _m^t(U^0)) ||_{\mathcal Y}^{} \right) \nonumber \\&\quad + \sup _{U\in \mathcal B^{ 2r_\phi }_0}||\mathrm D\Psi ^h_m(U) ||_{ \mathcal Y\rightarrow \mathcal Y}^{}\cdot E_m^n(U^0,h)\nonumber \\&\le \rho h^{p+1} m^{p+1-\ell } + (1+\sigma _\Psi h)E_m^n(U^0,h), \end{aligned}$$

for some $\rho > 0$. Due to (5.24), the second lines of (5.26a) and (5.26b) are valid as long as

5.27 $$\begin{aligned} \Phi _m^{nh}(U^0)+\theta ((\Psi ^h_m)^{n}(U^0)-\Phi _m^{nh}(U^0))\in \mathcal B_0^{2r_\phi }, \quad \theta \in [0,1], nh \le T, h\in [0,h_*]. \end{aligned}$$

Moreover the first supremum in (5.26b) is $O(m^{p+1-\ell })$ by Lemma 5.1, with *R* replaced by $r_\phi$. The second supremum in (5.26b) is $O(m^{p+1-\ell })$ by Lemma 5.2, with $\mathcal B^r_\ell$ replaced by $\mathcal B_\ell ^{r_\phi }$ (and *R* replaced by $2r_\phi \Lambda$).

Clearly $$E_m^0(U,h)=0$$, so

$$\begin{aligned} E_m^n(U,h)&\le \rho h^{p+1} m^{p+1-\ell }\frac{(1+\sigma _\Psi h)^n-1}{\sigma _\Psi h} \\&\le \frac{\rho }{\sigma _\Psi } h^pm^{p+1-\ell }\left( 1+\frac{n\sigma _\Psi h}{n}\right) ^n \le \frac{\rho }{\sigma _\Psi } h^pm^{p+1-\ell }e^{n\sigma _\Psi h}. \end{aligned}$$

Choosing $m(h) = h^{-p/(p+1)}$ we see that for $nh\le T$, $h \in [0,h_*]$,

5.28 $$\begin{aligned} \Vert (\Psi ^h_m)^n(U^0) - \Phi ^{nh}_m(U^0) \Vert _{\mathcal Y} \le \frac{\rho }{\sigma _\Psi }\mathrm e^{\sigma _\Psi T} h^{p}m ^{p+1-\ell } = C\mathrm e^{\sigma _\Psi T}h^{ \ell p/(p+1) }. \end{aligned}$$

Using (5.28) we can ensure that for $nh\le T$, $h \in [0,h_*]$

5.29 $$\begin{aligned} \Vert (\Psi ^h_m)^n(U^0) - \Phi ^{nh}_m(U^0) \Vert _{\mathcal Y} \le r_\phi \end{aligned}$$

by possibly reducing $h_*>0$, and hence that (5.27) holds.

*Step 3 *(*Projection error of numerical trajectory*) We now estimate the global projection error of the numerical method. We will prove that for $m(h) = h^{-p/(p+1)}$, $nh\le T$, $h \in [0,h_*]$,

5.30 $$\begin{aligned} \Vert (\Psi ^h)^n(U^0)-(\Psi _{m(h)}^h)^n(U^0)\Vert _{\mathcal Y} = \mathcal O( m^{-\ell } ) \end{aligned}$$

uniformly for initial data $U^0$ satisfying (5.20).

We first establish the required regularity of the numerical trajectory of the projected system: To bound the $\mathcal Y_{\ell }$-norm of the Galerkin truncated numerical trajectory $(\Psi ^h_{m(h)})^n(U^0)$ note that for $m=m(h) = h^{-p/(p+1)}$, $nh\le T$, $h \in [0,h_*]$, with $h_*$ small enough such that $m(h_*) \ge m_*$, we have

5.31 $$\begin{aligned} \Vert (\Psi _{m(h)}^h)^n(U^0)\Vert _{\mathcal Y_\ell }&\le \Vert (\Psi ^h_m)^n(U^0) - \Phi ^{nh}_m(U^0) \Vert _{\mathcal Y_\ell }+ \Vert \Phi ^{nh}_m(U^0) \Vert _{\mathcal Y_\ell } \nonumber \\&\le m^\ell \Vert (\Psi ^h_m)^n(U^0) - \Phi ^{nh}_m(U^0) \Vert _{\mathcal Y}+r_\phi \nonumber \\&\le m^\ell (C\mathrm e^{\sigma _\Psi T }m^{p+1-\ell } h^{p}) +r_\phi \le C\mathrm e^{\sigma _\Psi T }+r_\phi \le r_\psi \end{aligned}$$

for some $r_\psi >0$. Here $r_\phi$ is as in (5.22) and we used (2.4) in the second line and (5.28) in the third line.

To prove (5.30) let

$$\begin{aligned} e^j(U^0) = (\Psi ^h)^j(U^0) -(\Psi ^h_m)^j( U^0) \end{aligned}$$

be the truncation error at time $jh \le T$. Then for $(n+1)h\le T$,

5.32 $$\begin{aligned} e^{n+1}(U^0)&= (\Psi ^h \circ (\Psi ^h)^n)(U^0) -(\Psi ^h\circ (\Psi ^h_m)^n)( U^0) \nonumber \\&\quad + (\Psi ^h \circ (\Psi ^h_m)^n)(U^0) -(\Psi ^h_m\circ (\Psi ^h_m)^n)( U^0). \end{aligned}$$

By Theorem 4.3, with *r* replaced by $2r_\psi$ [and consequently *R* by $4r_\psi \Lambda$, see (3.6b)] we have

5.33 $$\begin{aligned} \Psi _m \in \mathcal C_{{\text {b}}}^1(\mathcal B_{0}^{2r_\psi }; \mathcal Y). \end{aligned}$$

By (5.24), with $\Psi _m$ replaced by $\Psi$ and the supremum taken over $\mathcal B_{0}^{2r_\psi }$, using (5.33) we get from (5.32) for $n\ge 1$, $h\in [0,h_*]$ and $(n+1)h\le T$ that

5.34 $$\begin{aligned} \Vert e^{n+1}(U^0) \Vert _{\mathcal Y}&\le \sup _{\theta \in [0,1] } \Vert \mathrm D\Psi ^h( (\Psi ^h_m)^n + \theta ((\Psi ^h)^n -(\Psi ^h_m)^n )(U^0) )\Vert _{ \mathcal Y\rightarrow \mathcal Y}\Vert e^n(U^0)\Vert _{\mathcal Y} \nonumber \\&\quad + \Vert e^1((\Psi ^h_m)^n(U^0))\Vert _{\mathcal Y} \nonumber \\&\le \sup _{\Vert U\Vert _{\mathcal Y_\ell } \le 2r_\psi } \Vert \mathrm D\Psi ^h(U)\Vert _{ \mathcal Y\rightarrow \mathcal Y}\Vert e^n(U^0)\Vert _{\mathcal Y} + \Vert e^1((\Psi ^h_m)^n(U^0))\Vert _{\mathcal Y} \nonumber \\&\le (1+\sigma _\Psi h)\Vert e^n(U^0)\Vert _{\mathcal Y} + h \mathcal O(m^{-\ell }), \end{aligned}$$

where $m=m(h)$, with order constant uniformly in all $U^0$ satisfying (5.20), as long as

5.35 $$\begin{aligned} (\Psi ^h_m)^n(U^0) + \theta ((\Psi ^h)^n(U^0) -(\Psi ^h_m)^n(U^0) ) \in \mathcal B_0^{ 2r_\psi }, \quad \theta \in [0,1]. \end{aligned}$$

Here we used that for $U \in \mathbb P_m\mathcal Y$,

$$\begin{aligned} e^1(U) = h\mathsf b^T({\text {id}}-h\mathsf aA)^{-1}\left( \left( \mathbb P_m( B(W(U,h))-B(W_m(U,h)) \right) + \mathbb Q_m B(W(U,h)) \right) , \end{aligned}$$

so that for $U \in \mathcal B_{\ell }^{r_\psi } \cap \mathbb P_m\mathcal Y$, $h\in [0,h_*]$, by (4.5c) (with *r* replaced by $r_\psi$ and *R* by $2r_\psi \Lambda$)

5.36 $$\begin{aligned} \Vert e^1(U)\Vert _{\mathcal Y}&\le h \Vert \mathsf b\Vert \Lambda ( M'\Vert W(U,h)-W_m(U,h)\Vert _{\mathcal Y^s} +\Vert \mathbb Q_m B(W(U,h))\Vert _{\mathcal Y^s}) \nonumber \\&\le h \Vert \mathsf b\Vert ( \Lambda M'\mathcal O(m^{-\ell }) + \mathcal O(m^{-\ell }) ) = h \mathcal O(m^{-\ell }), \end{aligned}$$

where $m=m(h)$ and $M'=M_0'[2r_\psi \Lambda ]$. In the last inequality of (5.36) we used that

5.37 $$\begin{aligned} \Vert \mathbb Q_m B(W(U,h))\Vert _{\mathcal Y^s} \le m^{-\ell } M = \mathcal O( m^{-\ell }), \end{aligned}$$

where $$M=M_0[2r_\psi \Lambda ]$$.

From (5.34) we deduce for $nh\le T$, $h\in [0, h_*]$ and all $U^0$ satisfying (5.20) that

5.38 $$\begin{aligned} \Vert e^n(U^0)\Vert _{\mathcal Y}&\le (1+\sigma _\Psi h)^{n-1}\Vert e^1(U^0)\Vert _{\mathcal Y} + \frac{1}{\sigma _\Psi h}\left( (1+\sigma _\Psi h)^{n-1}-1\right) h\mathcal O(m^{-\ell }) \nonumber \\&\le \exp (\sigma _\Psi T)(\Vert e^1(U^0)\Vert _{\mathcal Y} + \mathcal O(m^{-\ell })) = \mathcal O(m^{-\ell }), \end{aligned}$$

with $m=m(h)$. Here (5.36) does not apply to $\Vert e^1(U^0)\Vert _\mathcal Y$ because in general $U^0 \notin \mathbb P_m \mathcal Y$. But from (4.5d) we see that $\Vert e^1(U^0)\Vert _\mathcal Y= O(m^{-\ell })$. By choosing a possibly bigger $m_*$ (and, by virtue of $m=h^{-p/(p+1)}$, a smaller $h_*$) we can achieve that $\Vert e^n(U^0)\Vert _{\mathcal Y} \le r_\psi$ so that the required condition (5.35) is satisfied. This proves (5.30).

Hence, (4.3b), (5.28) and (5.30) prove that

5.39 $$\begin{aligned} E^n(U^0,h) = E_m^n(U^0,h) + \mathcal O(m^{-\ell }) = \mathcal O( h^{ p\ell /( p+1) } ) \end{aligned}$$

for $nh\le T$, $h\in [0, h_*]$ and $U^0$ satisfying (5.20). $\square$

#### *Example 5.4*

(*Cubic nonlinear Schrödinger equation in* $\mathbb R^3$) We now consider a cubic nonlinear Schrödinger equation in $\mathbb R^3$

5.40 $$\begin{aligned} \mathrm iu_t = \Delta u + |u|^2 u \end{aligned}$$

as in [13]. We rewrite it in the form (1.1) with $U = (u_1,u_2)$ where $u = u_1+\mathrm iu_2$ with

$$\begin{aligned} A= \left( \begin{array}{c@{\quad }c} 0 &{}\Delta \\ -\Delta &{} 0\\ \end{array} \right) ,\quad \text{ and }\quad B(U ) = (u_1^2 + u_2 ^2){u_2 \atopwithdelims ()-u_1}, \end{aligned}$$

cf. also Example 2.8, and consider it on $\mathcal Y=\mathcal H_{2}(\mathbb R^3;\mathbb R^2)$. By Lemma 2.9 a) the nonlinearity *B*(*U*) is analytic on $\mathcal Y$ and the same holds true on $\mathcal Y_\ell = D(A^\ell ) =\mathcal H_{2(\ell +1)}(\mathbb R^3,\mathbb R^2)$ where $\ell \ge 0$. In this case assumption (B) holds for $I=[0, L]$ and any $L>0$. If (5.40) is discretized by the implicit mid point rule and $U^0 \in \mathcal Y_1=\mathcal H_4$, then from Theorem 5.3 we obtain an order of convergence $\mathcal O(h^{2/3})$ in the $\mathcal H_2$-norm. In [13] a second order Strang type time discretization is used to discretize (5.40) and a better rate of convergence is observed, namely an order of convergence $\mathcal O(h)$ in the $\mathcal H_2$-norm for $U^0 \in \mathcal H_4$. This is due to the fact that the linear part of the evolution equation (1.1), i.e., $\dot{U} = AU$, is integrated exactly by this method. We plan to extend the methods of this paper to splitting and exponential integrators in future work.
